# Tumor-derived exosomes deliver the tumor suppressor miR-3591-3p to induce M2 macrophage polarization and promote glioma progression

**DOI:** 10.1038/s41388-022-02457-w

**Published:** 2022-09-09

**Authors:** Ming Li, Hao Xu, Yanhua Qi, Ziwen Pan, Boyan Li, Zijie Gao, Rongrong Zhao, Hao Xue, Gang Li

**Affiliations:** 1 Department of Neurosurgery, Qilu Hospital, Cheeloo College of Medicine and Institute of Brain and Brain-Inspired Science, Shandong University, Jinan, 250012 Shandong China; 2 Shandong Key Laboratory of Brain Function Remodeling, Jinan, 250012 Shandong China; 3 Department of Neurosurgery, The Affiliated Taian City Central Hospital of Qingdao University, Taian, 271000 Shandong China; 4 Department of Neurosurgery, The Affiliated Yantai Yuhuangding Hospital of Qingdao University, Yantai, 264000 Shandong China

**Keywords:** CNS cancer, Cancer microenvironment, Cell growth

## Abstract

Exosomes can selectively secrete harmful metabolic substances from cells to maintain cellular homeostasis, and complex crosstalk occurs between exosomes and tumor-associated macrophages (TAMs) in the glioma immune microenvironment. However, the precise mechanisms by which these exosome-encapsulated cargos create an immunosuppressive microenvironment remain unclear. Herein, we investigated the effect of glioma-derived exosomes (GDEs) on macrophage polarization and glioma progression. We performed sequencing analysis of cerebrospinal fluid (CSF) and tumor tissues from glioma patients to identify functional microRNAs (miRNAs). High levels of miR-3591-3p were found in CSF and GDEs but not in normal brain tissue or glial cells. Functionally, GDEs and miR-3591-3p significantly induced M2 macrophage polarization and increased the secretion of IL10 and TGFβ1, which in turn promoted glioma invasion and migration. Moreover, miR-3591-3p overexpression in glioma cell lines resulted in G2/M arrest and markedly increased apoptosis. Mechanistically, miR-3591-3p can directly target CBLB and MAPK1 in macrophages and glioma cells, respectively, and further activate the JAK2/PI3K/AKT/mTOR, JAK2/STAT3, and MAPK signaling pathways. In vivo experiments confirmed that macrophages lentivirally transduced with miR-3591-3p can significantly promote glioma progression. Thus, our study demonstrates that tumor-suppressive miR-3591-3p in glioma cells can be secreted via exosomes and target TAMs to induce the formation of an immunosuppressive microenvironment. Collectively, these findings provide new insights into the role of glioma exosomal miRNAs in mediating the establishment of an immunosuppressive tumor microenvironment and show that miR-3591-3p may be a valuable biomarker and that blocking the encapsulation of miR-3591-3p into exosomes may become a novel immunotherapeutic strategy for glioma.

## Introduction

Glioma is the most common and deadly primary intracranial malignancy of the adult central nervous system, and glioma—especially glioblastoma—has high heterogeneity and recurrence. Even with the current treatment strategies, including surgery, chemotherapy, radiotherapy, targeted therapy, etc., the median survival time and the overall 5-year survival rate remain poor after diagnosis [1–3].

Malignant glioma is recalcitrant to multiple therapeutic approaches because of its unique immunosuppressive tumor microenvironment (TME), which is composed of many different factors and many types of cells, including tumor cells and immune cells; extracellular matrix components; blood vessels; etc. Immunosuppressive cells include tumor-associated macrophages (TAMs), myeloid-derived suppressor cells (MDSCs), regulatory T cells (Tregs), etc.; among these cells, macrophages constitute the largest component of tumor-infiltrating immune cells and act as essential regulators during cancer progression [4–7]. Multiple studies [8–10], including our previous studies [11, 12], have shown that TAMs, including TAMs of the peripheral origin or microglial origin in the brain, are enriched in the glioma microenvironment and promote glioma progression. Recent studies have suggested that macrophage-targeted therapy is a novel and attractive approach for treating malignant tumors [13–16].

The activation of macrophages is an important event in the pathogenesis of various diseases. Macrophages are generally divided into two distinct subtypes: classically activated M1 macrophages and alternatively activated M2 macrophages. M1 macrophages secrete proinflammatory cytokines and phagocytose microorganisms, contributing to microbicidal and antitumor immunity; in contrast, M2 macrophages secrete anti-inflammatory factors and clear debris, contributing to angiogenesis and suppressing adaptive immunity, wound healing, and tissue repair [17, 18]. In the TME, infiltrated macrophages usually display the M2 phenotype and are termed TAMs. TAMs are critical for promoting tumor cell proliferation, metastasis, and angiogenesis. Moreover, tumor-secreted exogenous factors, such as exosomes, enhance the chemotaxis/migration of monocytes and the differentiation of monocytes into TAMs in the TME [11, 19, 20].

Exosomes are a type of extracellular vesicle (EV) with a diameter of approximately 30–150 nm and a lipid bilayer membrane. Exosomes are secreted by various cell types, including tumor cells, in biological fluids such as blood, cerebrospinal fluid (CSF), and urine [21, 22]. Exosomes were previously considered garbage processors, but an increasing number of studies are considering them to be critical for intercellular communication and to play essential roles in different physiological and pathological processes, including tumor-related processes. Exosomes contain several types of cargo, including proteins, lipids, enzymes, DNA fragments, messenger RNAs (mRNAs), microRNAs (miRNAs), and long noncoding RNAs (lncRNAs). In cancer, exosomes induce angiogenesis, cell migration and proliferation, inflammatory responses, immunosuppression, evasion of immune surveillance, and metastasis [23–26].

miRNAs, members of a class of ncRNAs with regulatory functions, are approximately 20–24 nt in length and are involved mainly in regulating gene expression at the posttranscriptional level by binding to the 3′‐untranslated regions (3′‐UTRs) of their target genes. As important regulatory molecules for biological processes, miRNAs participate in various regulatory pathways, including those mediating cell proliferation and apoptosis, TME formation, etc. [27]. The functional transfer of extracellular miRNAs has been convincingly demonstrated in numerous in vitro and in vivo studies, and exosomes are considered the main carriers of these miRNAs [28, 29].

In this study, our sequencing analysis showed that miR-3591-3p was expressed at lower levels in tumor tissues than in normal brain tissues (NBTs) but were present at higher levels in CSF exosomes. In addition, our data indicated that miR-3591-3p induced G2/M arrest in glioma cells and promoted their apoptosis and that these effects were achieved via inhibition of the MAP kinase (MAPK) pathway, suggesting that the tumor suppressor miR-3591-3p is selectively secreted by tumor cells. Moreover, exosomal miR-3591-3p was engulfed by macrophages and promoted their polarization toward an immunosuppressive phenotype by activating the JAK2/PI3K/Akt/mTOR and STAT3 pathways. Our study reveals a novel mechanism by which tumor cells evade immunosuppression via the secretion of specific tumor-suppressive molecules in exosomes to create a suppressive immune microenvironment that promotes tumor progression.

## Results

### Characterization of exosomes and PMA-treated THP-1 cells

First, we used ultracentrifugation to isolate GDEs from the supernatant of glioma cell lines (U118MG and U251). Observation of the extracted GDEs by TEM revealed vesicle-like structures with diameters ranging from 30-150 nm (Supplementary Fig. S1A). Subsequently, we performed particle size analysis of the extracted GDEs, confirming that the diameters of the particles were primarily 30-150 nm (Supplementary Fig. S1B). The western blot results showed that the extracted GDEs contained exosome-specific markers (TSG101 and CD63) but did not express an endoplasmic reticulum marker (Calnexin) (Supplementary Fig. S1C). In addition, human THP-1 cells were successfully differentiated into M0 macrophages by treatment with PMA (100 ng/mL) for 48 h (Supplementary Fig. S1F). In summary, we confirmed that we successfully extracted GDEs and that macrophages can phagocytose GDEs.

### Glioma cells promote M2 polarization of macrophages through secretion of exosomal miR-3591-3p

**Fig. 1:**
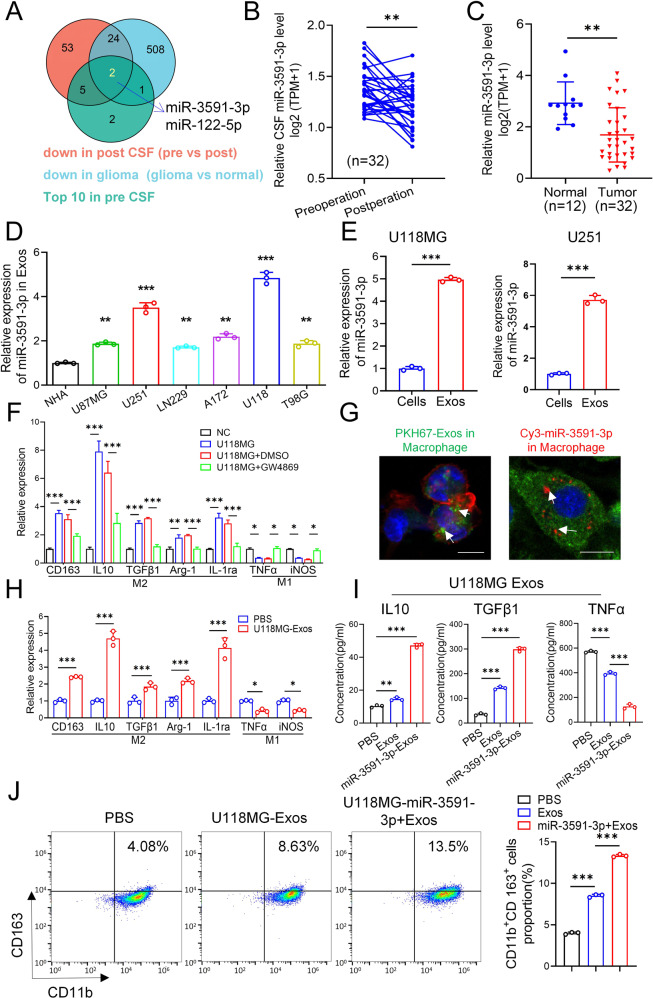
Glioma cell-derived exosomal miR-3591-3p promotes macrophage toward an M2-like phenotype. **A** Venn diagram exhibiting the overlap of the miRNAs in exosomes from CSF of glioma patients (preoperation vs. postoperation) and tissues (glioma tissues vs. normal brain tissues). **B** Preoperative and postoperative expression levels of miR-3591-3p in the CSF of glioma patients (*n* = 32). **C** The expression levels in tumor tissues (*n* = 32) and normal brain tissues (*n* = 12). **D** qPCR analysis of the expression levels of miR-3591-3p in the exosomes of different glioma cell lines and one normal cell line. **E** qPCR analysis of the expression levels of miR-3591-3p in the exosomes and cell lines of U118MG and U251. **F** qPCR analysis of the expression of M2 markers (CD163, IL10, TGFβ1, Arg-1, and IL1ra) and M1 markers (TNFα and iNOS) in PMA-pretreated THP-1 cells cocultured with U118MG cells (GW4869, exosome secretion inhibitor). **G** Representative immunofluorescence image showing the internalization of PKH67-labeled exosomes (green) and Cy3-labeled miR-3591-3p (red) by PMA-treated THP-1 cells. (scale bar,10 um). **H** The expression levels of M2 markers (CD163, IL10, Arg-1, TGFβ1, and IL1ra) and M1 markers (TNFα and iNOS) were determined by qRT–PCR in PMA-pretreated THP-1 cells treated with U118MG exosomes. **I** ELISAs were used to quantify the expression of cytokines (IL10, TGFβ, TNFα) in PMA-treated THP-1 cells cocultured with exosomes derived from U118MG cells transfected with miR-3591-3p mimics. **J** Flow cytometry assay was applied to analyze CD11b^+^CD163^+^ macrophages treated by exosomes derived from U118MG cells, and quantification was performed. Data are shown as the mean ± SD of three independent experiments. (*p < 0.05; **p < 0.01; ***p < 0.001).

To identify functional miRNAs involved in gliomagenesis and glioma progression, we analyzed the expression profiles of miRNAs in 32 glioma patient tumor tissues, preoperative CSF (pre-CSF), and postoperative CSF (post-CSF), and 8 normal brain tissues using RNA sequencing. We then intersected these 3 datasets (downregulated in post-CSF, FC > 1.5 and *p* < 0.05; downregulated in tumor tissues, FC > 1.5 and *p* < 0.05; and 10 most highly expressed in pre-CSF) to identify candidate miRNAs (miR-3591-3p and miR-122-5p) (Fig. 1A). Numerous studies have been conducted on miR-122-5p and have indicated that it plays an important role in various diseases [30, 31]. However, the regulatory effect of miR-3591-3p on tumor progression has rarely been studied. Therefore, here, we focused on miR-3591-3p. Further data analysis revealed that in 32 paired clinical CSF samples, the expression level of miR-3591-3p in post-CSF was significantly lower than that in pre-CSF (Fig. 1B). Moreover, the expression level was lower in tumor tissues than in normal brain tissues (Fig. 1C), which was an important result. To explore miR-3591-3p expression in exosomes, we assessed its expression level in exosome of six human glioma cell lines and normal human astrocytes (NHAs) and found that exosomes derived from all tested glioma cell lines contained high levels of miR-3591-3p (Fig. 1D). The two cell lines with the highest expression (U118MG and U251) were selected for this study. We next evaluated the amount of miR-3591-3p in exosomes and these two cell lines and found that miR-3591-3p was enriched primarily in exosomes. To explore the effect of glioma cells on macrophages in the TME, we cocultured glioma cells with PMA-induced THP-1 cells (THP-1-M*φ*) in a Transwell system in vitro (Supplementary Fig. S1E). We measured the levels of M2 and M1 macrophage-associated phenotypic markers in the different groups by qRT–PCR. The levels of M2 markers in macrophages cocultured with glioma cells were significantly increased, while those of M1 markers were decreased; treatment with GW4869 (an exosome release inhibitor) reversed the above effects (Fig. 1F, Supplementary Fig. S1D). These data suggest that glioma cells induce M2 polarization of macrophages in an exosome-dependent manner. To confirm that macrophages can engulf GDEs and miR-3591-3p, we cocultured PKH67 (green fluorescence)-labeled GDEs or Cy3 (red fluorescence)-conjugated miR-3591-3p with macrophages and found that the macrophages internalized the exosomes as well as exosome-encapsulated miR-3591-3p, as assessed by confocal microscopy (Fig. 1G). Next, to verify whether GDEs can regulate M2 macrophage polarization, we treated macrophages with PBS or GDEs. qRT–PCR analysis revealed that compared with PBS, GDEs increased the expression of M2 markers while reducing the expression of M1 markers (Fig. 1H, Supplementary Fig. S2A). In addition, we measured the levels of cytokines (IL10, TGFβ1, and TNFα) in the supernatants of macrophages by ELISA. Compared with PBS, GDEs markedly increased the secretion of the immune tolerance-associated cytokines (IL10 and TGFβ1) while dramatically decreasing the inflammatory cytokine secretion (TNF-α). Furthermore, glioma cells were transfected with the miR-3591-3p mimic or the corresponding NC vector, and exosomes released from miR-3591-3p mimic-transfected glioma cells (miR-3591-3p-Exos) were isolated and used to treat macrophages. The ELISA results showed that IL10 and TGFβ1 were further elevated, but the secretion of TNF-α was further reduced in the supernatants of macrophages treated with miR-3591-3p-Exos compared with the supernatants of macrophages treated with exosomes or PBS alone (Fig. 1I, Supplementary Fig. S2B). Consistent with this finding, flow cytometric analysis showed that the proportion of CD11b^+^CD163^+^ cells was significantly higher in the miR-3591-3p-Exos group than in the groups treated with exosomes or PBS (Fig. 1J, Supplementary Fig. S2C). In summary, these data suggest that miR-3591-3p can be encapsulated and secreted in GDEs, thereby inducing macrophage polarization toward the M2 phenotype.

### MiR-3591-3p promotes M2 macrophage polarization in vitro and in vivo

**Fig. 2:**
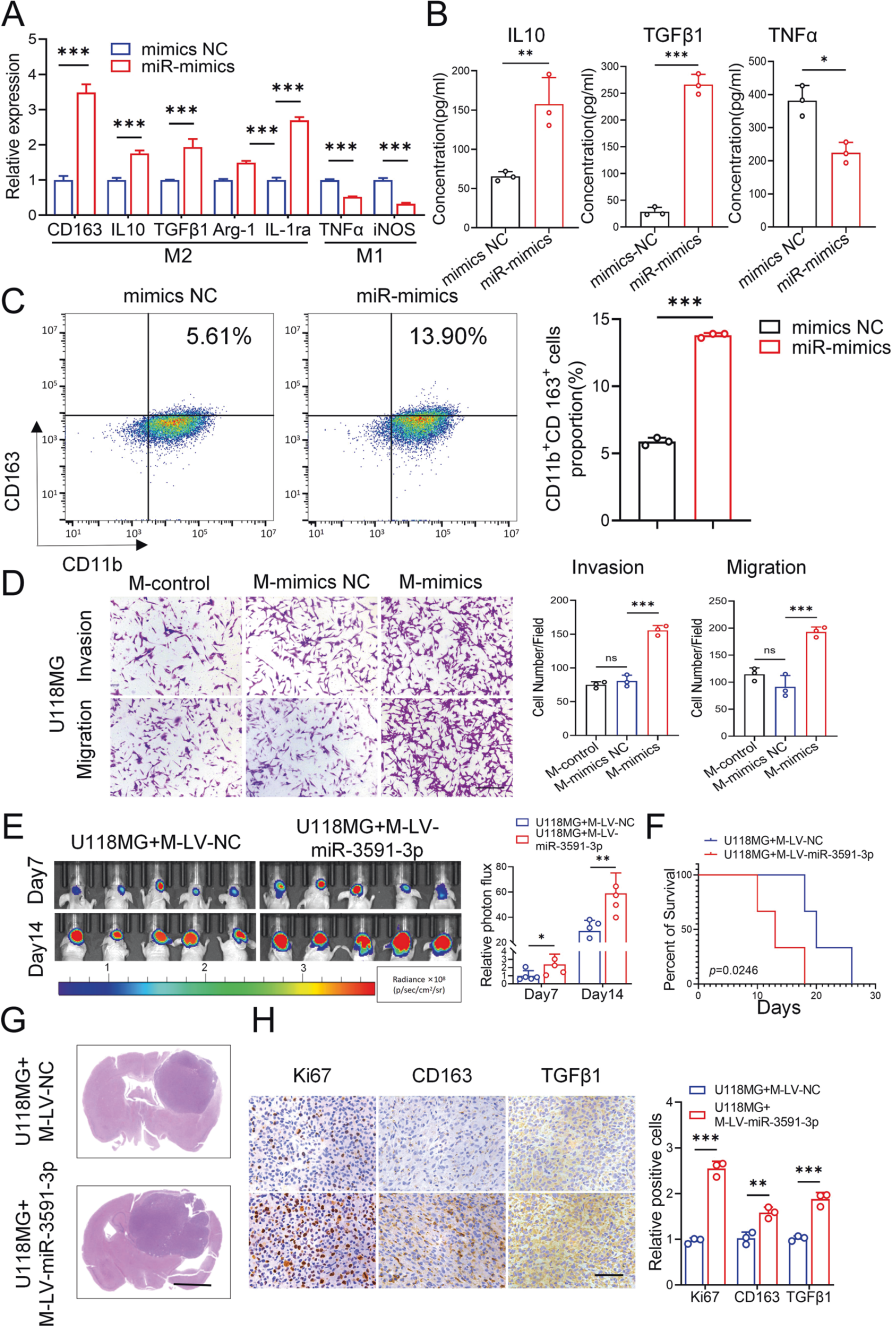
miR-3591-3p induced M2 macrophage polarization and promoted the progression of glioma cells in vitro and in vivo. **A** qRT–PCR analysis revealed that miR-3591-3p mimics could promote M2 macrophage polarization. **B** The secreted levels of cytokines (IL10, TGFβ1, and TNFα) were measured by ELISA in PMA-treated macrophages, which were transfected with miR-3591-3p mimics. **C** Representative flow cytometry plots of CD11b^+^ CD163^+^ macrophages. **D** Invasion and migration capacity of U118MG cocultured with conditioned macrophages were tested using transwell assays (PMA-treated THP-1 cells were transfected with miR-3591-3p mimics). Representative images of invaded and migrated cells are shown (scale bar, 100 μm). **E** In vivo bioluminescent imaging assay of tumor burden in xenograft nude mice bearing U118MG with macrophages pretreated with LV-miR-3591-3p or LV-NC. Representative images and relative photon flux on day 7 and day 14 postimplantation are shown (*n* = 5 of each group). **F** Survival proportion Kaplan–Meier curves of animals in each study group (*n* = 10 of each group). **G** HE staining of representative tumor tissues from two groups (scale bar, 2.5 mm). **H** Representative images and quantification of the expression of Ki67, CD163, and TGFβ1 using immunohistochemistry (scale bar, 100 μm). Data are shown as the mean ± SD of three independent experiments. (*p < 0.05; **p < 0.01; ***p < 0.001).

Subsequently, we investigated the role of miR-3591-3p in macrophage polarization. We transfected macrophages with the miR-3591-3p mimic, miR-3591-3p inhibitor, and the corresponding NCs. The qRT–PCR results revealed that the miR-3591-3p mimic increased the expression of M2 markers and decreased the expression of M1 markers (Fig. 2A), while the miR-3591-3p inhibitor had the opposite effects (Supplementary Fig. S2D). ELISAs showed that overexpression of miR-3591-3p promoted IL10 and TGFβ1 secretion and suppressed TNFα secretion, whereas inhibition of miR-3591-3p resulted in the opposite effects (Fig. 2B, Supplementary Fig. S2E). Additionally, the flow cytometry results showed that the number of CD11b^+^CD163^+^ cells was significantly increased after miR-3591-3p overexpression but decreased after miR-3591-3p knockdown (Fig. 2C, Supplementary Fig. S2F). Numerous studies have reported that tumor-derived exosome-induced M2 polarization of macrophages can promote tumor progression [11, 19]. Therefore, we investigated whether M2 macrophage polarization induced by exosomal miR-3591-3p could also enhance the invasive ability of glioma cells. To this end, glioma cells were cocultured with exogenous miR-3591-3p mimic-pretreated macrophages. Transwell assays showed that the numbers of migrated and invaded cells were significantly increased in the groups of U118MG and U251 cells cocultured with miR-3591-3p mimic-pretreated macrophages compared with the corresponding control groups (Fig. 2D. Supplementary Fig. S2H). Consistent with this observation, GSEA showed enrichment of TGFβ receptor signaling in EMT (Supplementary Fig. S2G). The above results demonstrated that overexpression of miR-3591-3p can induce M2 macrophage polarization, while M2-type macrophages can simultaneously enhance the invasive and migratory abilities of glioma cells. To further verify the above conclusion in vivo, glioma cells and macrophages infected with lentivirus overexpressing miR-3591-3p or miR-NC were simultaneously implanted intracranially into nude mice to establish an orthotopic xenograft model. In vivo bioluminescence imaging showed that nude mice implanted with glioma cells and macrophages with high expression of miR-3591-3p had stronger bioluminescence signals (Fig. 2E) and shorter overall survival times (Fig. 2F). In addition, HE staining showed that the tumors formed from macrophages transduced with miR-3591-3p-expressing lentivirus were larger than those in the control group (Fig. 2G). The IHC results showed that tumors formed from miR-3591-3p-overexpressing macrophages exhibited higher Ki67, CD163, and TGFβ1 expression levels (Fig. 2H). Collectively, our data indicate that miR-3591-3p can induce M2 macrophage polarization to establish an immunosuppressive TME, thereby contributing to the malignant progression of glioma.

### MiR-3591-3p induces M2 macrophage polarization by targeting CBLB

**Fig. 3:**
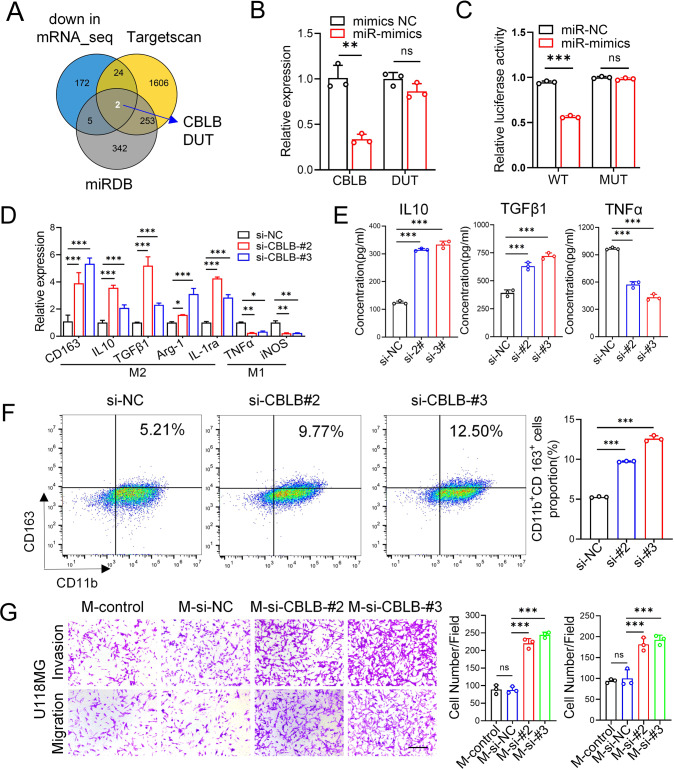
CBLB is the direct target of miR-3591-3p in macrophages. **A** Venn diagram showing the two predicted miR-3591-3p target genes in three databases (down in mRNA_seq, FC > 1.5, *p* <0.05; Targetscan; miRDB). **B** The expression of the two predicted genes was detected by qPCR analysis. **C** Relative luciferase activities of the CBLB luciferase reporters (WT and MUT) were performed in macrophages. **D** qRT–PCR was performed for M2 markers (CD163, IL10, TGFβ1, Arg-1, and IL1ra) and M1 markers (TNFα, iNOS) in macrophages transfected with si-CBLB or si-NC. **E** The expression levels of cytokines (IL10, TGFβ, TNFα) in macrophage transfected with si-CBLB or si-NC were detected by ELISA. **F** Flow cytometry assay was used to analyze CD11b+CD163+macrophages transfected with si-CBLB or si-NC, and quantification was performed. **G** Transwell assay was applied to determine the invasion and migration of U118MG cells cocultured with macrophages transfected with si-NC or si-CBLB. Representative images (scale bar, 100 μm) and quantification are shown. Data are shown as the mean ± SD of three independent experiments. (*p < 0.05; **p < 0.01; ***p < 0.001).

We further explored the mechanism by which tumor-derived exosomal miR-3591-3p induces M2 macrophage polarization. To determine the target of miR-3591-3p in macrophages, the differential expression of mRNAs in macrophages transfected with miR-NC and the miR-3591-3p mimic was analyzed by RNA sequencing (Supplementary Fig. S3A). We intersected the significantly downregulated genes (FC > 1.5, *p* < 0.05) with the potential target genes of miR-3591-3p predicted by the online bioinformatics tools TargetScan and miRDB (Fig. 3A). Two target genes were selected as candidates; these genes were further validated by qRT–PCR, which showed that the expression level of CBLB decreased more significantly than that of DUT (Fig. 3B). Next, to verify whether CBLB is a direct target of miR-3591-3p, the binding site for CBLB in miR-3591-3p was predicted and is shown in Supplementary Fig. S3B. Then, we performed a luciferase reporter assay and found that luciferase activity was markedly reduced in the group cotransfected with the miR-3591-3p mimic and CBLB-WT but was not significantly changed in the CBLB-MUT-transfected group, indicating that miR-3591-3p can directly target CBLB (Fig. 3C). To explore the relationship of CBLB with the immune response and immune microenvironment, we quantified tumor-infiltrating immune cells in glioma tissues from transcriptomic data by single-sample gene set enrichment analysis (ssGSEA). We classified glioma tissue samples in a cohort from The Cancer Genome Atlas (TCGA) into the “high infiltration” and “low infiltration” groups according to the number of infiltrating immune cells in the tissues. In most samples in the high infiltration group, the CBLB expression level was lower than the median expression level among all samples (Supplementary Fig. S3G). Furthermore, the results of the Tumor Immune Estimation Resource (TIMER) database analysis confirmed that the infiltrating levels of M2-type macrophages were negatively correlated with CBLB expression in both GBM and Lower-grade glioma (LGG) (Supplementary Fig. S3F). These results imply that CBLB is closely related to the infiltrating of TAMs. We then carried out subsequent experiments to explore the role of CBLB in the phenotypic conversion of macrophages. The CBLB knockdown efficiency was verified by western blotting (Supplementary Fig. S3C). qRT–PCR demonstrated that CBLB knockdown significantly increased the expression levels of M2 markers but decreased the expression levels of M1 markers (Fig. 3D). ELISA showed increased secretion of IL10 and TGFβ1 and reduced secretion of TNFα when CBLB was knocked down (Fig. 3E). Similarly, flow cytometric analysis revealed that CBLB knockdown significantly increased the proportion of CD11b^+^CD163^+^ cells (Fig. 3F). To further clarify whether knockdown of CBLB in macrophages can affect the migration/invasion abilities of glioma cells, we used a co-culture system for Transwell assays, which showed that the number of invaded or migrated cells cocultured with macrophages was clearly increased after knockdown of CBLB (Fig. 3G, Supplementary Fig. S3D). Taken together, these results suggest that miR-3591-3p can directly target CBLB, induce M2 macrophage polarization, and promote glioma progression.

### MiR-3591-3p promotes M2 macrophage polarization in a manner dependent on CBLB and the JAK2/PI3K/Akt/mTOR and JAK2/STAT3 pathway activation

**Fig. 4:**
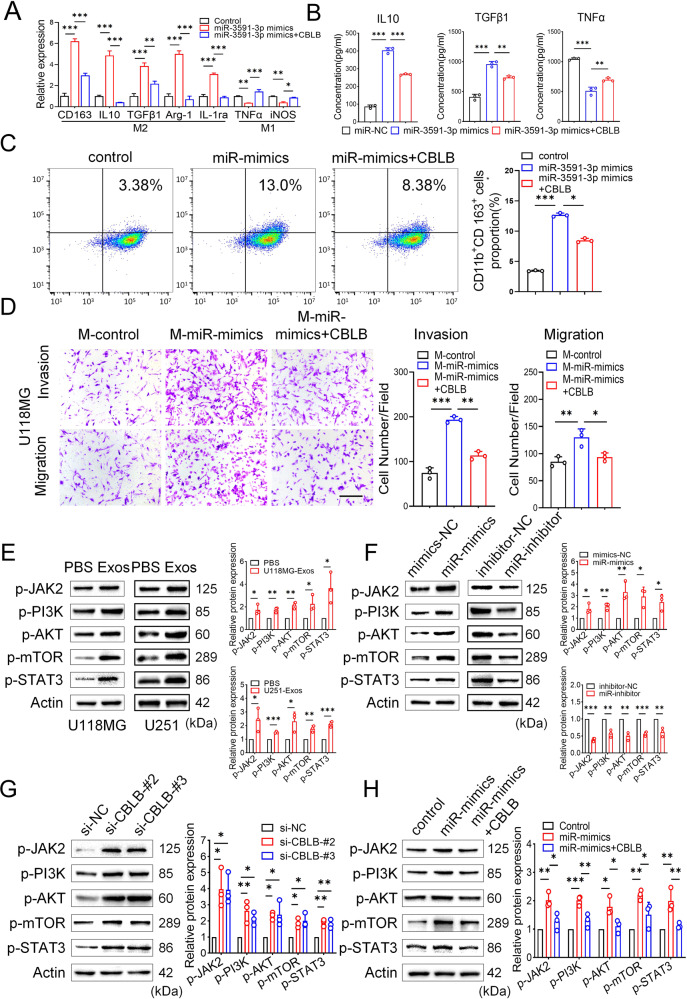
miR-3591-3p promotes macrophage toward M2 phenotype via downregulation of CBLB expression and activation of the JAK2/PI3K/AKT/mTOR and JAK2/STAT3 signaling pathway. **A** Macrophages were transfected with plasmids of CBLB following miR-3591-3p transfection, and the expression levels of M2 markers and M1 markers were detected by qRT–PCR. **B** The expression of cytokines (IL10, TGFβ1, and TNFα) were determined in macrophages, which were transfected with miR-3591-3p mimics and CBLB encoding plasmids. **C** The CD11b^+^CD163^+^ cells of macrophages were measured using flow cytometry. Representative images and quantification are shown. **D** Transwell assay was applied to determine the invasion and migration of U118MG cells cocultured with macrophages transfected with miR-3591-3p mimics and CBLB encoding plasmids. Representative images (scale bar, 100 μm) and quantification are shown. **E** Protein levels of JAK2/PI3K/AKT/mTOR and JAK2/STAT3 pathways were measured in macrophages cocultured with exosomes derived from glioma cells. **F** Protein levels of the two above pathways were detected following transfection of miR-3591-3p mimics or inhibitors in macrophages. **G** Proteins levels of the two above pathways were measured in macrophages transfected with si-CBLB or si-NC. **H** Western blot analysis of the two pathways protein levels after the indicated treatment. The intensity of protein bands was quantified by densitometry. Macrophages: PMA-pretreated THP-1 cells. Data are shown as the mean ± SD of three independent experiments. (*p < 0.05; **p < 0.01; ***p < 0.001).

To investigate whether CBLB is essential for M2 macrophage polarization, we performed a series of functional rescue experiments. The CBLB overexpression efficiency was validated by western blotting (Supplementary Fig. S4A). The qRT–PCR, ELISA, and flow cytometry experiments showed that the promotive effect of miR-3591-3p on M2 macrophage polarization was reversed or partially reversed after CBLB overexpression (Fig. 4A–C). Moreover, overexpression of CBLB reversed the promotive effect of miR-3591-3p-overexpressing macrophages on glioma cell invasion and migration (Fig. 4D, Supplementary Fig. S4D). These results suggest that CBLB is indispensable for the promotive effect of miR-3591-3p on macrophage polarization. Next, to further explore the underlying mechanism by which miR-3591-3p promotes macrophage polarization by targeting CBLB, we used bioinformatic methods to analyze the differentially expressed genes in the sequencing data. GO enrichment analysis showed that the differentially expressed genes were significantly enriched in the following biological functions: myeloid activation involved in the immune response, myeloid leukocyte-mediated immunity, and negative regulation of phagocytosis (Supplementary Fig. S4B). In addition, the differentially expressed genes were identified as being associated with glioma and tumor immunity via DisGeNET gene-disease association analysis (Supplementary Fig. S4C). Furthermore, as an E3 ubiquitin ligase, CBLB can promote the ubiquitination and degradation of different protein substrates. Analysis of the UbiBrowser database (http://ubibrowser.ncpsb.org/) [32] revealed that JAK2 is a direct substrate of CBLB (Supplementary Fig. S4E). Consistent with this finding, evidence has indicated that JAK2 is ubiquitinated and degraded in a manner regulated by CBLB [33]. In addition, the JAK2/PI3K/AKT/mTOR and STAT3 signaling pathways have previously been demonstrated to be associated with the phenotypic conversion of macrophages and may act by influencing the metabolic pattern of the cells [20, 34–36]. Both GO analysis and GSEA showed that the differentially expressed genes were significantly enriched in processes related to cell metabolism (Supplementary Fig. S4B, Supplementary Fig. S4F). Therefore, we hypothesized that miR-3591-3p might promote M2 macrophage polarization via the JAK2/PI3K/Akt/mTOR and STAT3 signaling pathways. To test our hypothesis, we performed a series of western blot analyses and found that GDEs activated the JAK2/PI3K/Akt/mTOR and STAT3 pathways (Fig. 4E); the same effect was observed when miR-3591-3p was overexpressed in macrophages (Fig. 4F). Moreover, the phosphorylation of related proteins in this pathway was enhanced when CBLB was knocked down (Fig. 4G). In addition, the rescue experiments showed that CBLB overexpression partially attenuated the effect of miR-3591-3p overexpression (Fig. 4H). Taken together, our data suggest that CBLB is essential for miR-3591-3p-induced M2 macrophage polarization, which is achieved via activation of the JAK2/PI3K/AKT/mTOR and STAT3 pathways.

### MiR-3591-3p promotes cell cycle arrest and apoptosis in vitro and in vivo

**Fig. 5:**
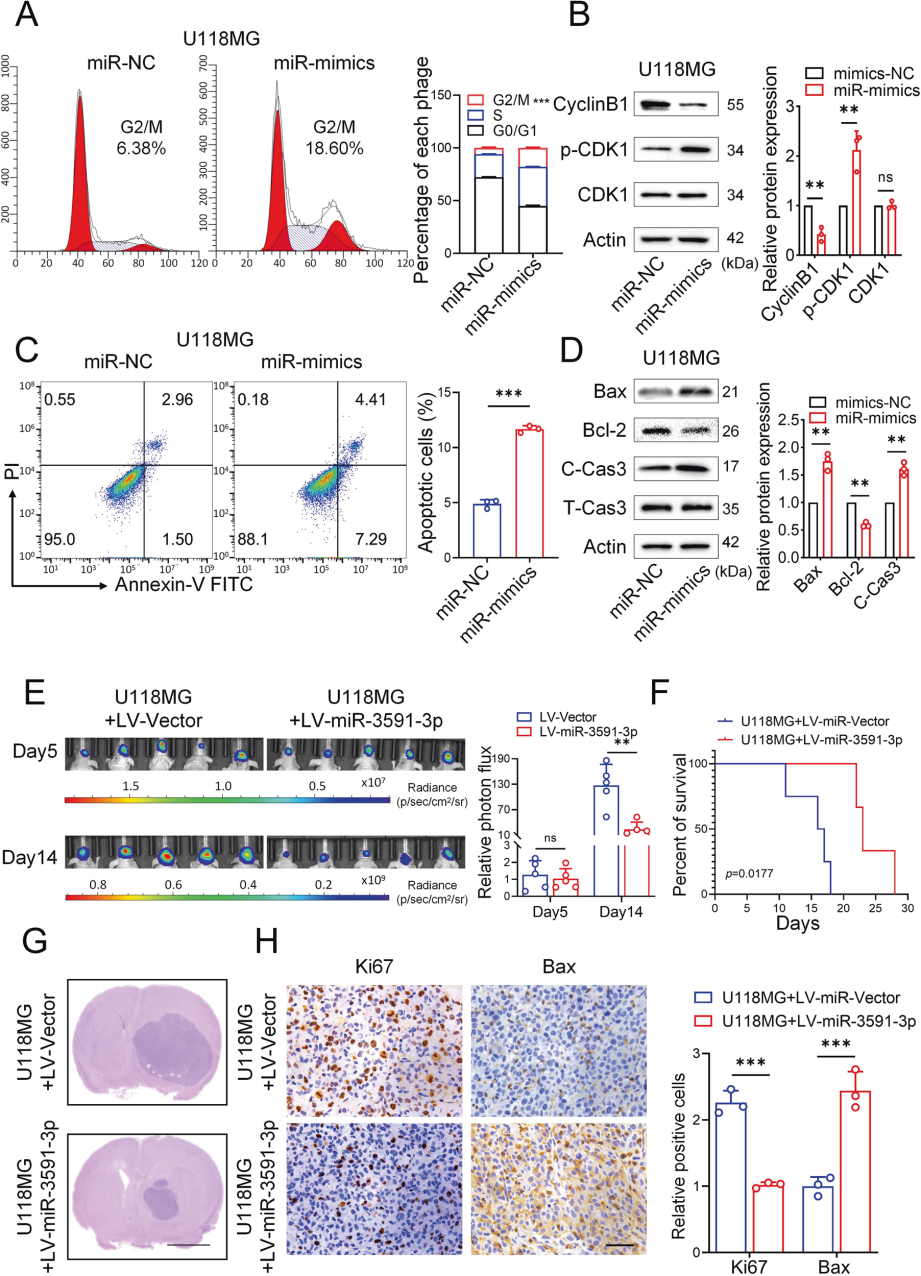
miR-3591-3p suppresses tumor growth in vitro and in vivo by blocking cell cycle progression. **A** Flow cytometry analysis of cell cycle phase is shown of U118MG cells transfected with miR-NC or miR-3591-3p mimics. **B** Western blots were used to detect the levels of G2/M-related proteins. **C** Representative flow cytometry plots of cell apoptosis and quantitative analysis are shown. **D** Western blot analysis of apoptosis-associated proteins. **E** In vivo bioluminescent imaging assay of tumor burden in xenograft nude mice bearing U118MG pretreated with LV-miR-3591-3p or LV-NC. Representative images and quantifications are shown (*n* = 5 of each group). **F** Kaplan–Meier survival curves for overall survival in different groups (*n* = 10 of each group). **G** Representative HE staining of tumor tissues are shown from the two groups (scale bar, 2.5 mm). **H** Representative pictures of immunohistochemical staining of Ki67 and Bax are shown (scale bar, 100 μm). Data are shown as the mean ± SD of three independent experiments. (*p < 0.05; **p < 0.01; ***p < 0.001).

Our previous results showed that miR-3591-3p was enriched in exosomes and that the level of miR-3591-3p expression in glioma cells was significantly lower than that in normal glial cells (Fig. 1B, C). Therefore, we sought further to explore the role of miR-3591-3p in glioma cells. First, we transfected the miR-3591-3p mimic or miR-NC into glioma cells and performed transcriptome sequencing. As shown in Fig. S5E, GO analysis of the downregulated genes revealed that most of these genes were significantly enriched in the mitosis and cell cycle biological processes. Next, we overexpressed miR-3591-3p in glioma cells, and flow cytometric analysis revealed G2/M arrest in these cells (Fig. 5A, Supplementary Fig. S5A). In addition, we analyzed the expression of the G2/M-related proteins and found that CyclinB1 expression was decreased, but p-CDK1 expression was significantly increased (Fig. 5B, Supplementary Fig. S5B). In addition, we observed that the apoptosis rate was obviously increased when miR-3591-3p was overexpressed (Fig. 5C, Supplementary Fig. S5C). Similarly, the expression of the apoptosis-related proteins also exhibited a trend consistent with that mentioned above (Fig. 5D, Supplementary Fig. S5D). To further determine the function of miR-3591-3p in vivo, we stably overexpressed miR-3591-3p in fluorescein-labeled U118MG cells (generating LV-miR-3591-3p cells) and injected them into the brains of nude mice to establish an intracranial orthotopic xenograft model. Bioluminescence imaging showed that mice implanted with LV-miR-3591-3p cells had much lower luciferase signals than those bearing LV-vector cells (Fig. 5E) and had longer survival times (Fig. 5F). In addition, HE staining showed that the tumor volume was significantly reduced after overexpression of miR-3591-3p (Fig. 5G). The IHC results confirmed that the number of Ki67-positive cells was significantly decreased, but the number of Bax-positive cells was increased in the LV-miR-3591-3p group (Fig. 5H).

### miR-3591-3p targets MAPK1 and suppresses glioma growth via the MAPK signaling pathway

**Fig. 6:**
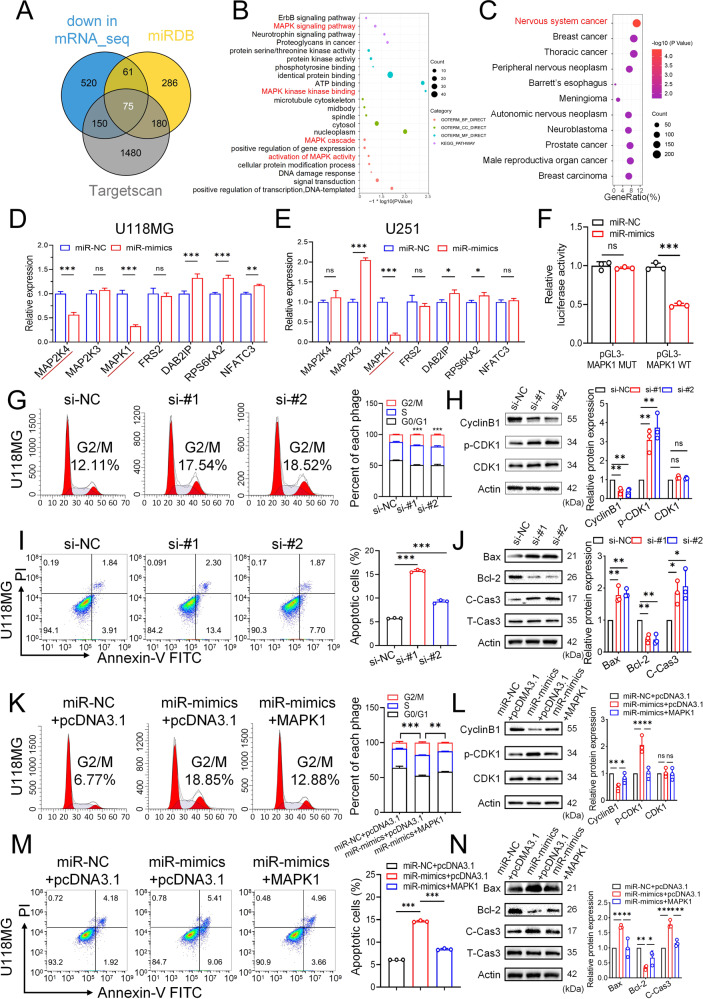
MAPK1 is the direct downstream target of miR-3591-3p in glioma cells. **A** Venn diagram showing the predicted miR-3591-3p target genes in three databases (down in mRNA_seq, FC > 1.5, *p* < 0.05; Targetscan; miRDB). **B** DO (Disease Ontology) enrichment analysis of differential expression genes in mRNA_seq. **C** GO and KEGG pathway enrichment analysis of the 75 overlapping genes. **D**, **E** The relative mRNA expression levels of the 7 MAPK signaling pathway-related genes were measured transfected with miR-mimics/NC in U118MG and U251 cell lines, respectively. **F** Relative luciferase activity was detected by dual-luciferase reporter assays. **G** Representative flow cytometry results of the cell cycle phase are shown in U118MG cells transfected with si-NC or si-MAPK1. **H** The expression of G2/M-related proteins was detected by western blot. **I** Representative flow cytometry results of apoptotic cells are shown in U118MG cells transfected with si-NC or si-MAPK1. **J** The expression of apoptosis-related proteins was detected by western blot in groups as indicated. **K** MAPK1 overexpressing plasmids were transfected following miR-3591-3p mimics transfection, and the cell cycle distributions were determined by flow cytometry in U118MG cells. **L** The expression of G2/M-related proteins was detected by western blot in groups as indicated. **M** MAPK1 overexpressing plasmids were transfected following miR-3591-3p mimics transfection, and the apoptotic cells were quantified by flow cytometry in U118MG cells. **N** Western blot assessment of the expressions of apoptosis-related proteins in groups as indicated. Data are shown as the mean ± SD of three independent experiments. (*p < 0.05; **p < 0.01; ***p < 0.001).

**Fig. 7:**
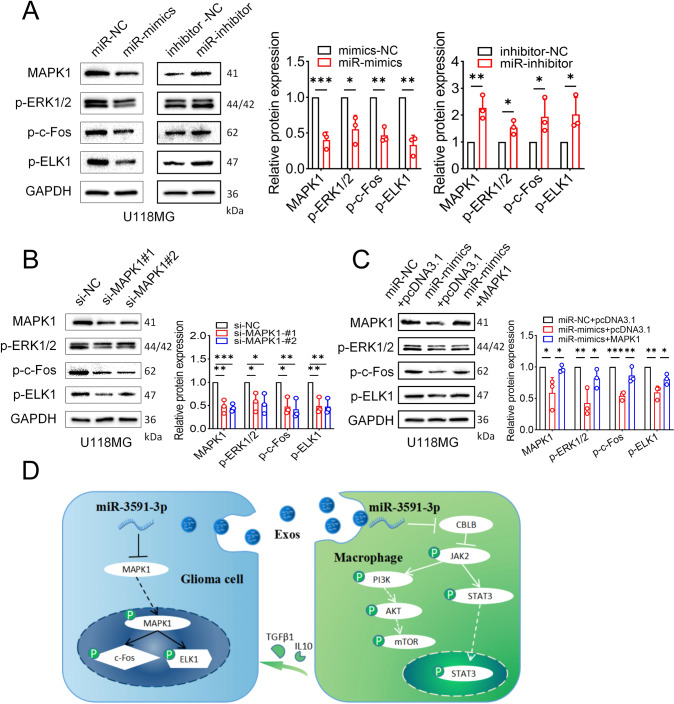
miR-3591-3p suppresses tumor via MAPK signaling pathway. **A** Western blot results for the expression of proteins in the MAPK signaling pathway in U118MG cells transfected with miR-NC, miR-3591-3p mimics, inhibitor-NC, and miR-3591-3p inhibitor. **B** Western blot results for the expression of proteins in the MAPK signaling pathway in U118MG cells transfected with si-NC or si-MAPK1. **C** MAPK1 overexpressing plasmids were transfected following miR-3591-3p mimics transfection, and the MAPK signaling pathway-related proteins were detected by western blot in U118MG cells. The intensity of protein bands was quantified by densitometry. **D** Schematic diagram depicting the key assumptions and conclusions. Briefly, miR-3591-3p was excreted by glioma cells via exosomes and then taken up by macrophages. In macrophages, miR-3591-3p promotes macrophage to M2 phenotype by inhibiting the activity of CBLB and then activating JAK2/PI3K/AKT/mTOR and STAT3 pathways. On the other hand, since miR-3591-3p plays a tumor suppressor role in glioma cells, when it is excreted, the inhibitory effect on MAPK1 is relieved and thus promoting glioma progression by activating the MAPK pathway. Data are shown as the mean ± SD of three independent experiments. (*p < 0.05; **p < 0.01; ***p < 0.001).

To identify the targets of miR-3591-3p in glioma cells, we performed mRNA sequencing (mRNA-seq) to determine the differentially expressed mRNAs in glioma cells transfected with miR-3591-3p mimic and miR-NC. We then intersected the downregulated differentially expressed genes with the target genes predicted by the two online databases (TargetScan and miRDB) (Fig. 6A). A total of 75 overlapping genes were selected for further analysis. To gain more insight into the biological functions of these 75 genes, we performed KEGG and GO analyses and found that these genes were significantly enriched in the MAPK pathway (Fig. 6B). In addition, we utilized the DO database for gene-disease association analysis and found that the differentially expressed genes were significantly enriched in central nervous system cancers (Fig. 6C). Among the 7 candidate target genes, only MAPK1 was downregulated considerably by miR-3591-3p in the U118MG and U251 cell lines (Fig. 6D, E). The MAPK1 binding sites in miR-3591-3p are shown in Fig. S6A. To further determine whether miR-3591-3p directly binds to the 3′-UTR of MAPK1, we performed dual-luciferase assays in HEK-293-T cells. MiR-3591-3p markedly reduced luciferase activity in the MAPK1 WT groups but had no significant effect in the MAPK1 MUT groups (Fig. 6F). Next, to explore the role of MAPK1 in glioma cells, we knocked down MAPK1 using siRNAs. The MAPK1 knockdown efficiency was estimated by western blotting (Supplementary Fig. S6B). The flow cytometry results showed a G2/M phase arrest upon MAPK1 knockdown (Fig. 6G, Supplementary Fig. S6C). Western blotting was performed to detect G2/M-related proteins, and we found that CyclinB1 expression was significantly decreased, while p-CDK1 expression showed the opposite trend (Fig. 6H, Supplementary Fig. S6D). Apoptosis analysis showed that the number of apoptotic cells was significantly higher in the MAPK1 knockdown groups than in the control groups (Fig. 6I, Supplementary Fig. S6E). Next, the activity of apoptosis-related proteins, including the anti-apoptotic protein Bcl-2 and the pro-apoptotic proteins Bax and cleaved caspase-3, was examined. Western blot analysis showed that siRNA transfection enhanced the expression of pro-apoptotic proteins but inhibited the expression of anti-apoptotic proteins (Fig. 6J, Supplementary Fig. S6F). To further elucidate whether the suppression of glioma progression is mediated through the miR-3591-3p–MAPK1 axis, we overexpressed MAPK1 in glioma cells and performed gain-of-function experiments to verify whether MAPK1 was able to rescue the phenotypic effects of miR-3591-3p. G2/M arrest was abrogated by overexpression of MAPK1 (Fig. 6K, Supplementary Fig. S6G). In addition, the percentage of apoptotic cells was significantly restored when MAPK1 was overexpressed (Fig. 6M, Supplementary Fig. S6I). Moreover, the expression levels G2/M-related and apoptosis-related proteins were then analyzed, and the results were consistent with the aforementioned flow cytometry data (Fig. 6L, N; Supplementary Fig. S6H, J). The results of our previous bioinformatic analysis indicated that miR-3591-3p might function through the MAPK pathway (Fig. 6B). Therefore, we further investigated the effect of miR-3591-3p and MAPK1 on the MAPK pathway. Indeed, the p-ERK, p-c-Fos, and p-ELK1 levels were decreased in glioma cells transfected with the miR-3591-3p mimic while transfection of the miR-3591-3p inhibitor led to the opposite trend (Fig. 7A, Supplementary Fig. S7A). Additionally, MAPK1 knockdown significantly decreased the levels of p-ERK, p-c-Fos, and p-ELK1 (Fig. 7B, Supplementary Fig. S7B). Furthermore, the decreases in the levels of p-ERK, p-c-Fos, and p-ELK1 were partially reversed after MAPK1 overexpression (Fig. 7C, Supplementary Fig. 7C). Collectively, the above findings demonstrate that the inhibitory effect of miR-3591-3p on glioma cells is mediated via direct targeting of MAPK1 and inhibition of the MAPK pathway.

## Discussion

Accumulating evidence has shown that miRNAs play essential roles in tumorigenesis and tumor progression. The TME is a complex dynamic system orchestrated by intercellular communication and is responsible for tumor progression and metastasis. Numerous studies have shown that exosomal cargos can mediate the interactions between tumor cells and other cells and contribute to the formation of an immunosuppressive microenvironment. In addition, our previous study showed that circular RNAs (circRNAs) or miRNAs in GDEs could promote M2 polarization of TAMs [11] or induce the immunosuppressive function of MDSCs [29]. However, in the current study, we report that miR-3591-3p exerts differential effects on glioma cells and macrophages.

We performed miRNA sequencing on CSF exosomes and paired glioma tissues and normal brain tissues and found that some miRNAs had high expression in the CSF of glioma patients and low expression in tumor tissues. Our results demonstrated that overexpressing miR-3591-3p can inhibit glioma proliferation by targeting MAPK1. MiR-3591-3p can be secreted by glioma cells via exosomes, which alleviates MAPK pathway inhibition and promotes glioma progression. In addition, the secreted miR-3591-3p can be phagocytosed by macrophages and lead to polarization toward an immunosuppressive phenotype by inhibiting CBLB expression.

Recent reports indicate that exosome release plays a crucial role in maintaining cellular homeostasis. This task relies on the selective removal of harmful substances such as DNA, proteins, and ncRNAs from cells [37–40]. Consistent with this idea, our study shows that glioma cells can secrete tumor-suppressive miR-3591-3p via exosomes. Interestingly, however, we found that glioma cells can establish a suppressive TME by sorting the tumor suppressor miR-3591-3p into exosomes and being phagocytosed by macrophages. We discovered that miR-3591-5p could target MAPK1 in glioma cells and inhibit the activity of the MAPK pathway, thereby inhibiting tumor proliferation. MAPK1 encodes a member of the MAPK family. MAPKs, also called extracellular signal-regulated kinases (ERKs), are integration points of diverse biochemical signals and are involved in various cellular processes, such as proliferation, differentiation, transcriptional regulation, and development [41]. Activation of MAPK1 requires its phosphorylation by upstream kinases. Upon activation, MAPKs translocate into the nucleus of stimulated cells, where they phosphorylate their downstream proteins. The MAPK signaling pathway plays a vital role in supporting cancer survival and proliferation [42]. We found that when MAPK1 was knocked down, the phosphorylation of its downstream proteins was inhibited, resulting in the inhibition of glioma proliferation. Considering the critical role of MAPK1 in glioma proliferation, finding new strategies to inhibit its expression is essential for improving the clinical outcome of glioma patients. Although reports indicate that tumor cells can sort miRNAs into exosomes to promote tumor development, the effects of miRNAs packaged into exosomes on the TME remain incompletely defined. To date, the secretion of miR-3591-3p by tumor cells via exosomes and its potential role in the TME has not been reported. We hypothesized that miR-3591-3p in exosomes could counteract antitumor immune activity by promoting macrophage phenotypic conversion. We reveal for the first time that miR-3591-3p can be transferred into macrophages via exosomes and target CBLB, which activates the JAK2/PI3K/Akt/mTOR and STAT3 pathways in macrophages and facilitates the polarization of M2‐type macrophages. CBLB encodes an E3 ubiquitin ligase that promotes proteasome-mediated protein degradation by transferring ubiquitin from an E2 ubiquitin-conjugating enzyme to a substrate. Previous studies have shown that CBLB participates in regulating immune responses in lymphocytes, including T cells, B cells, and natural killer (NK) cells [43–45]. However, the mechanism by which CBLB functions in macrophages remains undefined. In the present study, we found that knockdown of CBLB activated the JAK2/PI3K/Akt/mTOR and STAT3 pathways and promoted macrophage polarization toward the M2 phenotype.

M2-polarized TAMs can secrete large amounts of cytokines into the TME. They play a vital role in regulating tumor growth, migration, etc., and are essential cytokines in cancer cell biology, contributing to the development and progression of several human cancers [46]. In the present study, we showed that macrophages induced to polarize toward the M2 phenotype by glioma cell-derived exosomal miR-3591-3p could promote glioma invasion and migration by secreting IL10 and TGFβ, contributing to sustained crosstalk between tumor cells and TAMs and creating a malignant microenvironment promoting tumor formation. Thus, our results suggest that miR-3591-3p plays a dual role in glioma development: tumor cells can selectively release tumor-suppressive miR-3591-3p via exosomes, and these exosomes can be internalized by macrophages, promoting their M2 polarization, which in turn promotes glioma progression. However, the mechanism by which miR-3591-3p is selectively encapsulated in exosomes is warranted in further study. Our findings not only identify a specific miRNA marker in CSF liquid biopsy of patients with glioma but also offer a novel perspective on evasion of immunosuppression in glioma and provide new insight into the treatment of glioma.

In summary, we found that exosomal miR-3591-3p can facilitate macrophage polarization toward the M2 phenotype by targeting CBLB to activate the JAK2/PI3K/Akt/mTOR and STAT3 pathways, which in turn promotes glioma progression. Moreover, miR-3591-3p acts as a tumor suppressor by inhibiting the MAPK pathway in glioma cells and is transferred into the TME to contribute to forming an immunosuppressive TME. Therefore, these findings help improve the current understanding of exosome biology and provide a new direction for the treatment of glioma. Exosomal miR-3591-3p may be a potential clinical biomarker for glioma.

## Materials and methods

### Patient sample collection

Glioma tissue and CSF samples (*n* = 32) from patients undergoing surgical resection in the Department of Neurosurgery, Qilu Hospital of Shandong University, between November 2017 and October 2019 were included. Normal brain tissue samples (*n* = 12) were obtained from patients who underwent partial resection of brain tissues due to intracerebral hemorrhage or traumatic brain injury. The study was approved by the Clinical Research Ethics Committee of Qilu Hospital of Shandong University and was performed according to the Declaration of Helsinki. In addition, all patients provided informed consent.

### Cell culture

The human glioma cell lines U118MG and U251 and the monocytic leukemia cell line THP-1 were purchased from the Cell Bank of the Chinese Academy of Sciences. U118MG and U251 cells were cultured in Dulbecco’s modified Eagle’s medium (DMEM; Thermo Fisher Scientific, USA) supplemented with 10% fetal bovine serum (FBS; Thermo Fisher Scientific), 100 U/mL penicillin, and 100 μg/mL streptomycin. THP-1 cells were cultured in RPMI 1640 medium (Thermo Fisher Scientific) supplemented with 10% FBS, 100 U/mL penicillin, and 100 μg/mL streptomycin. THP-1 cells were incubated with 100 ng/mL phorbol-12-myristate-13-acetate (PMA; Sigma–Aldrich, USA) for 24 h to induce differentiation into macrophages in vitro. For exosome co-culture, exosomes (1 μg/mL) were added to the culture medium of recipient cells. All cell lines were authenticated by short tandem repeat (STR) profiling and cultured in an incubator with 5% CO_2_ at 37 °C.

### Exosome isolation and identification

Glioblastoma multiforme (GBM) cell lines were cultured in DMEM supplemented with 10% exosome-depleted FBS. Exosomes were isolated from cell culture supernatants using several sequential centrifugation and ultracentrifugation steps as previously described [29]. The ultrastructure and size of EVs were assessed using transmission electron microscopy (TEM) and a ZetaView instrument (Particle Metrix, Germany), respectively.

### Exosome and miRNA uptake assay

To monitor exosomal trafficking, exosomes isolated from the culture medium were labeled with a PKH67 fluorescent cell linker kit (Sigma–Aldrich, USA). After PKH67 staining, the exosomes were washed in PBS, centrifuged at 100,000 × *g* for 20 min at 4 °C, and resuspended in PBS. Then, the PKH67-labeled exosomes were incubated with macrophages for 24 h. To monitor the trafficking of EVs containing miRNAs, glioma-derived exosomes (GDEs) were isolated from the culture medium of glioma cells transfected with Cy3-labeled miR-3591-3p and cocultured with macrophages. Nuclei and the cytoskeleton were stained with DAPI and FITC-phalloidin, respectively. Finally, the uptake of exosomes and miRNA was examined by confocal fluorescence microscopy.

### Cell transduction

The miR-3591-3p mimics/inhibitors and the CBLB and MAPK1 small interfering RNAs (siRNAs) were purchased from GenePharma (Shanghai, China). Lipofectamine^TM^ 3000 Transfection Reagent (Thermo Fisher Scientific, USA) was used to transfect the miRNA mimics, inhibitors, siRNAs, and CBLB/MAPK1 overexpression plasmids into cells according to the manufacturer’s instructions. Twelve hours after transfection, the cell culture medium was replaced with a fresh medium for subsequent experiments. The miR-3591-3p overexpression and control lentiviruses were purchased from Genechem (Shanghai, China). For stable overexpression of miR-3591-3p in glioma cells, cells were infected with lentivirus containing the miR-3591-3p mimic or miR negative control (miR-NC) according to the manufacturer’s protocol. All sequences used in this study are available in Supplementary Table S2.

### RNA extraction and quantitative reverse transcription-polymerase chain reaction (qRT–PCR)

Total cellular RNA was extracted using TRIzol (Invitrogen, USA) according to the manufacturer’s protocol. Exosomal RNA was extracted using a SeraMir^TM^ Exosome RNA Extraction Kit (System Biosciences, USA) after isolation of exosomes using Exoquick (System Biosciences). RNA (1 μg per sample) was reverse transcribed into complementary DNA (cDNA) using PrimeScript RT Reagent (TaKaRa, Japan). qRT–PCR was performed in a Roche LightCycler 480 System using TB Green Premix Ex Taq^TM^ (Takara, Japan). All reactions were performed in triplicate, and miR-3591-3p expression relative to U6 expression and gene expression relative to GAPDH expression were quantified by the comparative cycle threshold (2^−ΔΔCt^) method. The sequences of the primers are listed in Supplementary Table S1.

### Western blotting

Total cellular protein and exosomal protein were extracted using radioimmunoprecipitation assay (RIPA) lysis buffer containing protease inhibitor cocktail (Sigma–Aldrich). Protein extracts were separated by 8-12% sodium dodecyl sulfate-polyacrylamide gel electrophoresis (SDS–PAGE) and transferred onto polyvinylidene fluoride (PVDF) membranes (Millipore, Billerica, USA). After blocking with 5% bovine serum albumin (BSA; Sigma–Aldrich, St. Louis, USA), membranes were incubated first with primary antibodies (Supplementary Table S3) overnight at 4 °C and then with horseradish peroxidase-conjugated secondary antibodies for 1 h at room temperature. Protein bands were visualized by enhanced chemiluminescence (ECL; Millipore, Bedford, USA), while protein band intensities were analyzed with ImageJ software.

### Flow cytometry

To detect macrophage surface markers, anti-CD163-PE (BD Biosciences, USA) and anti-CD11b-APC (eBioscience, USA) antibodies were used to stain cells at 4 °C for 30 min. Isotype controls were run in parallel. After washing, a BD Accuri C6 flow cytometer (BD Biosciences) was used to determine the proportion of CD11b^+^CD163^+^ macrophages. To detect cell cycle arrest, glioma cells were collected 48 h after transfection and fixed with ice-cold 70% ethanol overnight. Then, the cells were incubated with 0.5 mL of propidium iodide (BD Biosciences) in the dark at room temperature for 15 min and were finally analyzed with a BD Accuri C6 flow cytometer (BD Biosciences) and ModFit software (Verity Software House, USA). An Annexin V-FITC apoptosis detection kit (BD Biosciences) was utilized to evaluate apoptosis according to the manufacturer’s instructions. In brief, cells were stained with Annexin V-FITC and propidium iodide. After incubation at room temperature in the dark for 15 min, apoptosis was quantified using a BD Accuri C6 flow cytometer (BD Biosciences) within 1 h of staining.

### ELISA

Cell culture medium was collected 72 h after the indicated treatment. Secretion of IL10, TGFβ1, and TNFα was examined using a Quantikine ELISA Kit (Proteintech, USA) according to the manufacturer’s instructions.

### Dual-luciferase reporter assay

The dual-luciferase reporter plasmids (CBLB wild-type (WT)/mutant (MUT) and MAPK1 WT/MUT) were designed and synthesized in the vector pGL3 (GenePharma, Shanghai, China). HEK-293T cells were cotransfected with the WT or MUT plasmids and the miR-3591-3p/mimic using Lipofectamine 3000 (Thermo Fisher Scientific, USA). After 48 h of transfection, cell lysates were harvested, and luciferase reporter assays were conducted using a Dual-Luciferase Reporter Assay Kit (Promega, USA) according to the manufacturer’s instructions. Firefly luciferase activity was normalized to Renilla luciferase activity.

### Transwell assays

Glioma cell invasion and migration were evaluated using 24-well Transwell plates (Corning, USA) with an 8.0 μm pore polycarbonate membrane. The upper surface of the membrane was coated with Matrigel (50 µL/well, BD Biosciences, USA) for the invasion assay but not for the migration assay. Tumor cells (5 × 10^4^) were seeded in the upper chamber with 200 µL of serum-free DMEM medium, while the bottom chamber contained 600 µL of medium supplemented with 10% FBS. After incubation for 24 h, the cells remaining in the upper chamber were removed, and the cells that had invaded the membrane or migrated to the bottom chamber were fixed with paraformaldehyde and stained with 0.1% crystal violet.

### Bioinformatic analysis

The online miRNA prediction databases TargetScan (http://www.targetscan.org/vert_72/) and miRDB (http://mirdb.org/) were used to identify putative targets of miR-3591-3p. Gene Ontology (GO) and Kyoto Encyclopedia of Genes and Genomes (KEGG) pathway enrichment analyses of differentially expressed genes were performed with the cluster profile R package. We used the cluster profile package to determine the statistical enrichment of differentially expressed genes in Reactome pathways, Disease Ontology (DO) pathways, and DisGeNET pathways. The local version of the Gene Set Enrichment Analysis (GSEA) tool (http://www.broadinstitute.org/gsea/index.jsp) was applied for GSEA. A *p*-value less than 0.05 indicated significant enrichment with differentially expressed genes.

### Immunohistochemistry

Immunohistochemical (IHC) staining was conducted using the streptavidin-peroxidase-biotin method. In brief, brain tissues of xenografted nude mice were collected, formalin-fixed, and paraffin-embedded. The specimens were sectioned at a thickness of 4 μm. Then, the sections were blocked with 10% normal goat serum (Gibco, USA) and incubated first with primary antibodies (Supplementary Table S3) at 4 °C overnight and then with a biotinylated secondary antibody at 37 °C for 30 min. Finally, signals were visualized with diaminobenzidine (DAB) solution, and the sections were counterstained with hematoxylin. Representative images were acquired using a microscope (Leica DM 2500, Germany). Positively stained cells were quantified using ImageJ software.

### Animal studies

All animal study procedures were approved by the Institutional Animal Care and Use Committee of Qilu Hospital. All 4-week-old male athymic nude mice were purchased from SLAC Laboratory Animal Center (Shanghai, China) and housed in a specific pathogen-free (SPF) environment. The miR-3591-3p and control lentiviral vectors were efficiently delivered into both U118MG cells and macrophages to establish stable miR-3591-3p-overexpressing cell lines and NC cell lines, respectively. To verify the effect of macrophages on glioma cells in vivo, luciferase-labeled U118MG cells (1 × 10^6^ cells per mouse) mixed with miR-3591-3p-overexpressing macrophages (2 × 10^5^ cells per mouse) were stereotactically injected into the brains of nude mice. To investigate the effect of miR-3591-3p on glioma cells in vivo, a total of 1 × 10^6^ luciferase-labeled glioma cells were injected into the brains of nude mice. Next, we randomly selected five mice per group and sacrificed them two weeks after implantation. The brains were fixed with paraformaldehyde for further study (hematoxylin and eosin (HE) and IHC staining). The remaining mice (10 per group) were retained until they died for survival analysis. Tumor volumes were measured and quantified by in vivo bioluminescence imaging with an IVIS Lumina Series III (PerkinElmer) after cell implantation.

### Statistical analysis

All statistical analyses were performed using Statistical Product and Service Solutions (SPSS 22.0) and GraphPad Prism 8 (GraphPad Software Inc., CA, USA) software. The Kaplan–Meier method was used to construct survival curves, and log-rank tests were used to assess the significance of the differences. Student’s t-test or one-way analysis of variance (ANOVA) was used for all other data comparisons in SPSS 22.0 software. All data are presented as the mean ± standard deviation (SD) of at least three independent experiments. Data were visualized using GraphPad Prism 8, and *p* < 0.05 was considered statistically significant (NS, *p* > 0.05; *p < 0.05; **p < 0.01; ***p < 0.001).

## Supplementary information

Supplementary Figures

Table S1

Table S2

Table S3

## Data Availability

All data used in the current study are available from the corresponding author on reasonable request. The microRNA sequencing and transcriptome sequencing data have been uploaded with accession numbers CAR002339, PRJNA807450, and PRJNA807393.

## References

[1] Miller KD, Ostrom QT, Kruchko C, Patil N, Tihan T, Cioffi G (2021). Brain and other central nervous system tumor statistics, 2021. CA Cancer J Clin.

[2] Ostrom QT, Patil N, Cioffi G, Waite K, Kruchko C, Barnholtz-Sloan JS (2020). CBTRUS statistical report: primary brain and other central nervous system tumors diagnosed in the United States in 2013-2017. Neuro Oncol.

[3] Janjua TI, Rewatkar P, Ahmed-Cox A, Saeed I, Mansfeld FM, Kulshreshtha R (2021). Frontiers in the treatment of glioblastoma: past, present and emerging. Adv Drug Deliv Rev.

[4] Aldape K, Brindle KM, Chesler L, Chopra R, Gajjar A, Gilbert MR (2019). Challenges to curing primary brain tumours. Nat Rev Clin Oncol.

[5] Dapash M, Hou D, Castro B, Lee-Chang C, Lesniak MS. The Interplay between Glioblastoma and Its Microenvironment. Cells. 2021;10:2257. 10.3390/cells10092257 PMC8469987 34571905

[6] Barthel L, Hadamitzky M, Dammann P, et al. Glioma: molecular signature and crossroads with tumor microenvironment. Cancer Metastasis Rev. 2022;41:53–75. 10.1007/s10555-021-09997-9 PMC8924130 34687436

[7] Chen D, Zhang X, Li Z, Zhu B (2021). Metabolic regulatory crosstalk between tumor microenvironment and tumor-associated macrophages. Theranostics.

[8] Liu T, Zhu C, Chen X, Wu J, Guan G, Zou C (2022). Dual role of ARPC1B in regulating the network between tumor-associated macrophages and tumor cells in glioblastoma. Oncoimmunology.

[9] Chen P, Zhao D, Li J, Liang X, Li J, Chang A (2019). Symbiotic macrophage-glioma cell interactions reveal synthetic lethality in PTEN-null glioma. Cancer Cell.

[10] Pombo Antunes AR, Scheyltjens I, Lodi F, Messiaen J, Antoranz A, Duerinck J (2021). Single-cell profiling of myeloid cells in glioblastoma across species and disease stage reveals macrophage competition and specialization. Nat Neurosci.

[11] Pan Z, Zhao R, Li B, Qi Y, Qiu W, Guo Q (2022). EWSR1-induced circNEIL3 promotes glioma progression and exosome-mediated macrophage immunosuppressive polarization via stabilizing IGF2BP3. Mol Cancer.

[12] Zhang Z, Xu J, Chen Z, Wang H, Xue H, Yang C (2020). Transfer of MicroRNA via macrophage-derived extracellular vesicles promotes proneural-to-mesenchymal transition in glioma stem cells. Cancer Immunol Res.

[13] Anderson NR, Minutolo NG, Gill S, Klichinsky M (2021). Macrophage-based approaches for cancer immunotherapy. Cancer Res.

[14] Xia Y, Rao L, Yao H, Wang Z, Ning P, Chen X (2020). Engineering macrophages for cancer immunotherapy and drug delivery. Adv Mater.

[15] Kang M, Lee SH, Kwon M, Byun J, Kim D, Kim C (2021). Nanocomplex-mediated in vivo programming to chimeric antigen receptor-M1 macrophages for cancer therapy. Adv Mater.

[16] Tzetzo SL, Abrams SI (2021). Redirecting macrophage function to sustain their “defender” antitumor activity. Cancer Cell.

[17] Murray PJ (2017). Macrophage polarization. Annu Rev Physiol.

[18] Yan S, Wan G (2021). Tumor-associated macrophages in immunotherapy. FEBS J.

[19] Xu J, Zhang J, Zhang Z, Gao Z, Qi Y, Qiu W (2021). Hypoxic glioma-derived exosomes promote M2-like macrophage polarization by enhancing autophagy induction. Cell Death Dis.

[20] Morrissey SM, Zhang F, Ding C, Montoya-Durango DE, Hu X, Yang C (2021). Tumor-derived exosomes drive immunosuppressive macrophages in a pre-metastatic niche through glycolytic dominant metabolic reprogramming. Cell Metab.

[21] Kalluri R, LeBleu VS. The biology, function, and biomedical applications of exosomes. Science. 2020;367:eaau6977. 10.1126/science.aau6977 PMC7717626 32029601

[22] He G, Peng X, Wei S, Yang S, Li X, Huang M (2022). Exosomes in the hypoxic TME: from release, uptake and biofunctions to clinical applications. Mol Cancer.

[23] Hosseini R, Sarvnaz H, Arabpour M, Ramshe SM, Asef-Kabiri L, Yousefi H (2022). Cancer exosomes and natural killer cells dysfunction: biological roles, clinical significance and implications for immunotherapy. Mol Cancer.

[24] Ma F, Vayalil J, Lee G, Wang Y, Peng G. Emerging role of tumor-derived extracellular vesicles in T cell suppression and dysfunction in the tumor microenvironment. J Immunother Cancer. 2021;9:e003217. 10.1136/jitc-2021-003217 PMC8513270 34642246

[25] Hoshino A, Kim HS, Bojmar L, Gyan KE, Cioffi M, Hernandez J (2020). Extracellular vesicle and particle biomarkers define multiple human cancers. Cell.

[26] Cheng J, Meng J, Zhu L, Peng Y (2020). Exosomal noncoding RNAs in Glioma: biological functions and potential clinical applications. Mol Cancer.

[27] O’Brien K, Breyne K, Ughetto S, Laurent LC, Breakefield XO (2020). RNA delivery by extracellular vesicles in mammalian cells and its applications. Nat Rev Mol Cell Biol.

[28] Vignard V, Labbe M, Marec N, Andre-Gregoire G, Jouand N, Fonteneau JF (2020). MicroRNAs in tumor exosomes drive immune escape in melanoma. Cancer Immunol Res.

[29] Qiu W, Guo X, Li B, Wang J, Qi Y, Chen Z (2021). Exosomal miR-1246 from glioma patient body fluids drives the differentiation and activation of myeloid-derived suppressor cells. Mol Ther.

[30] Zhou H, Sun F, Ou M, Zhang Y, Lin M, Song L (2021). Prior nasal delivery of antagomiR-122 prevents radiation-induced brain injury. Mol Ther.

[31] Wang Z, Wang X (2020). miR-122-5p promotes aggression and epithelial-mesenchymal transition in triple-negative breast cancer by suppressing charged multivesicular body protein 3 through mitogen-activated protein kinase signaling. J Cell Physiol.

[32] Li Y, Xie P, Lu L, Wang J, Diao L, Liu Z (2017). An integrated bioinformatics platform for investigating the human E3 ubiquitin ligase-substrate interaction network. Nat Commun.

[33] Lv K, Jiang J, Donaghy R, Riling CR, Cheng Y, Chandra V (2017). CBL family E3 ubiquitin ligases control JAK2 ubiquitination and stability in hematopoietic stem cells and myeloid malignancies. Genes Dev.

[34] Kaneda MM, Messer KS, Ralainirina N, Li H, Leem CJ, Gorjestani S (2016). PI3Kgamma is a molecular switch that controls immune suppression. Nature.

[35] Villarino AV, Kanno Y, O’Shea JJ (2017). Mechanisms and consequences of Jak-STAT signaling in the immune system. Nat Immunol.

[36] Yu H, Pardoll D, Jove R (2009). STATs in cancer inflammation and immunity: a leading role for STAT3. Nat Rev Cancer.

[37] Takahashi A, Okada R, Nagao K, Kawamata Y, Hanyu A, Yoshimoto S (2017). Exosomes maintain cellular homeostasis by excreting harmful DNA from cells. Nat Commun.

[38] Iguchi Y, Eid L, Parent M, Soucy G, Bareil C, Riku Y (2016). Exosome secretion is a key pathway for clearance of pathological TDP-43. Brain.

[39] Teng Y, Ren Y, Hu X, Mu J, Samykutty A, Zhuang X (2017). MVP-mediated exosomal sorting of miR-193a promotes colon cancer progression. Nat Commun.

[40] Chen C, Yu H, Han F, Lai X, Ye K, Lei S (2022). Tumor-suppressive circRHOBTB3 is excreted out of cells via exosome to sustain colorectal cancer cell fitness. Mol Cancer.

[41] Lavoie H, Gagnon J, Therrien M (2020). ERK signalling: a master regulator of cell behaviour, life and fate. Nat Rev Mol Cell Biol.

[42] Wang Y, Liu S, Yang Z, Algazi AP, Lomeli SH, Wang Y (2021). Anti-PD-1/L1 lead-in before MAPK inhibitor combination maximizes antitumor immunity and efficacy. Cancer Cell.

[43] Nguyen TTT, Wang ZE, Shen L, Schroeder A, Eckalbar W, Weiss A. Cbl-b deficiency prevents functional but not phenotypic T cell anergy. J Exp Med. 2021;218:e20202477. 10.1084/jem.20202477 PMC8117209 33974042

[44] Li X, Gong L, Meli AP, et al. Cbl and Cbl-b control the germinal center reaction by facilitating naive B cell antigen processing. J Exp Med. 2020;217:e20191537. 10.1084/jem.20191537 PMC7478728 32584413

[45] Guo X, Mahlakõiv T, Ye Q, et al. CBLB ablation with CRISPR/Cas9 enhances cytotoxicity of human placental stem cell-derived NK cells for cancer immunotherapy. J Immunother Cancer. 2021;9:e001975. 10.1136/jitc-2020-001975 PMC7986888 33741730

[46] Franklin RA, Liao W, Sarkar A, Kim MV, Bivona MR, Liu K (2014). The cellular and molecular origin of tumor-associated macrophages. Science.

