# Supplementary material for: Tumor-derived exosomes deliver the tumor suppressor miR-3591-3p to induce M2 macrophage polarization and promote glioma progression
Source: Oncogene. 2022 Sep 9;41(41):4618–32. doi: 10.1038/s41388-022-02457-w (PMC9546774; doi:10.1038/s41388-022-02457-w)
Supplement: Supplementary file 1 — Supplementary Figures [file 41388_2022_2457_MOESM1_ESM.docx]

**Supplementary Figures**

**
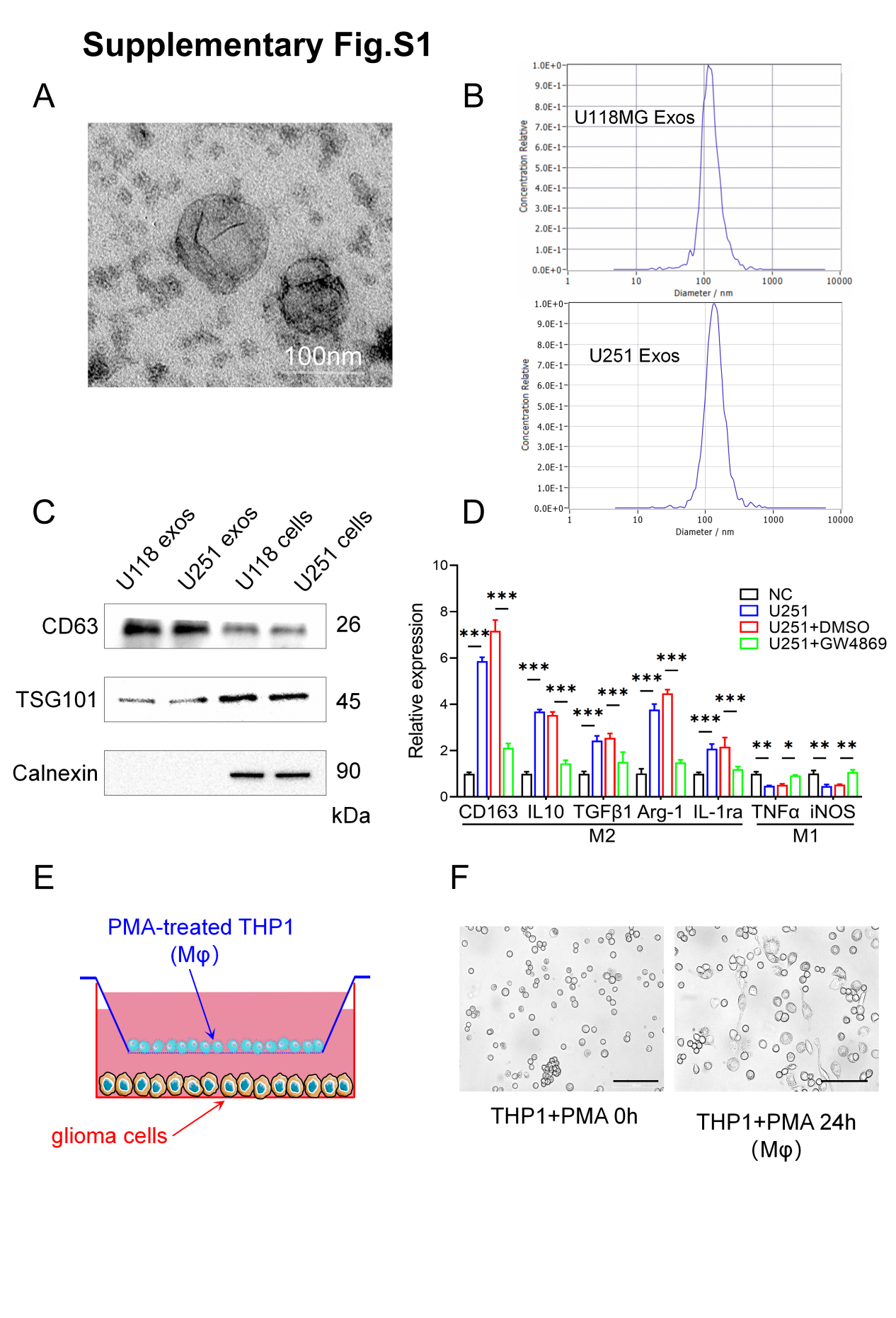
**

**Fig. S1 Characterization of glioma exosomes and PMA-treated THP1.** **A** Representative transmission electron microscope image of exosomes derived from U118MG and U251 cells. **B** Western blot analysis was performed to detect typical exosomal biomarkers (CD63, TSG101, and Calnexin) in exosomes derived from U118MG and U251 cell lines. **C** Representative nanoparticle tracking analysis of exosomes (U118MG, U251). **D** qPCR analysis of the expression of typical M2 markers (CD163, IL10, TGFβ1, Arg-1, IL1ra) and M1 markers (TNFα and iNOS) in PMA-pretreated THP-1 cells cocultured with U251 cells and cells depleted of exosomes by GW4869. **E** Schematic illustration of the co-culturing model for macrophages and glioma cells using a transwell chamber （pore size, 0.4 μm）. After PMA induction, THP1 (Mφ) were mono-cultured (NC) or co-cultured with untreated glioma cells (U118MG or U251), glioma cells treated with DMSO, or glioma cells treated with 5μM GW4869. **F** Representative image of macrophages derived from THP-1 cells treated with phorbol-12-myristate-13-acetate (PMA) (100ng/ml) for 24 h.

**
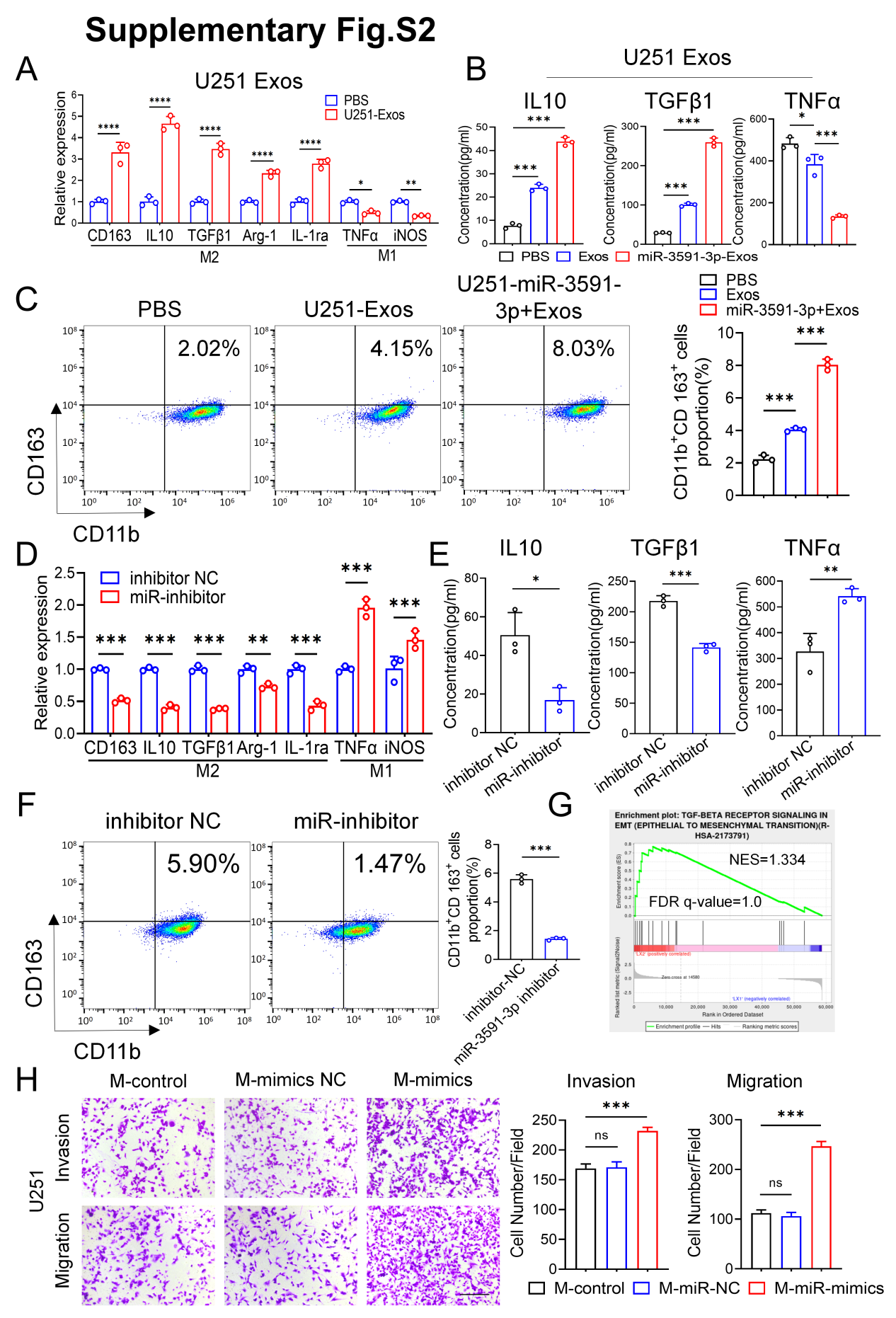
Fig.S2** **Exosomes derived from U251 cells promote macrophage toward an M2-like phenotype, and miR-3591-3p inhibitors reduce M2-like phenotype.** **A** The expression levels of M2 markers (CD163, IL-10, Arg-1, TGFβ1, and IL1ra) and M1 markers (TNFα and iNOS) were determined by qRT-PCR in PMA-pretreated THP-1 cells treated with U118MG exosomes. **B** ELISAs were used to measure the expression of cytokines (IL10, TGFβ, TNFα) in PMA-treated THP-1 cells cocultured with exosomes derived from U251 cells transfected with miR-3591-3p mimics. **C** Flow cytometry assay was applied to analyze CD11b^+^CD163^+^ macrophages treated by exosomes derived from U118MG cells, and quantification was performed. **D** qRT-PCR analysis revealed that miR-3591-3p inhibitors could suppress M2 macrophage polarization. **E** The secreted levels of cytokines (IL10, TGFβ1, and TNFα) were measured by ELISA in PMA-treated macrophages, which were transfected with miR-3591-3p inhibitors. **F** Flow cytometry was applied to measure CD11b^+^ CD163^+^ macrophages transfected with miR-3591-3p inhibitors. **G** Reactome datasets were used for GSEA analysis of mRNA_seq. It showed that differential expression genes in macrophages treated with miR-3591-3p mimics were enriched in TGFβ receptor signaling in EMT. **H** Invasion and migration capacity of U251 cocultured with conditioned macrophages were tested using transwell assays (PMA-treated THP1 cells were transfected with miR-3591-3p mimics). Representative images of invaded and migrated cells are shown (scale bar, 100 μm). Data are shown as the mean ± SD of three independent experiments. (**p* < 0.05; ***p* < 0.01; ****p* < 0.001).

**
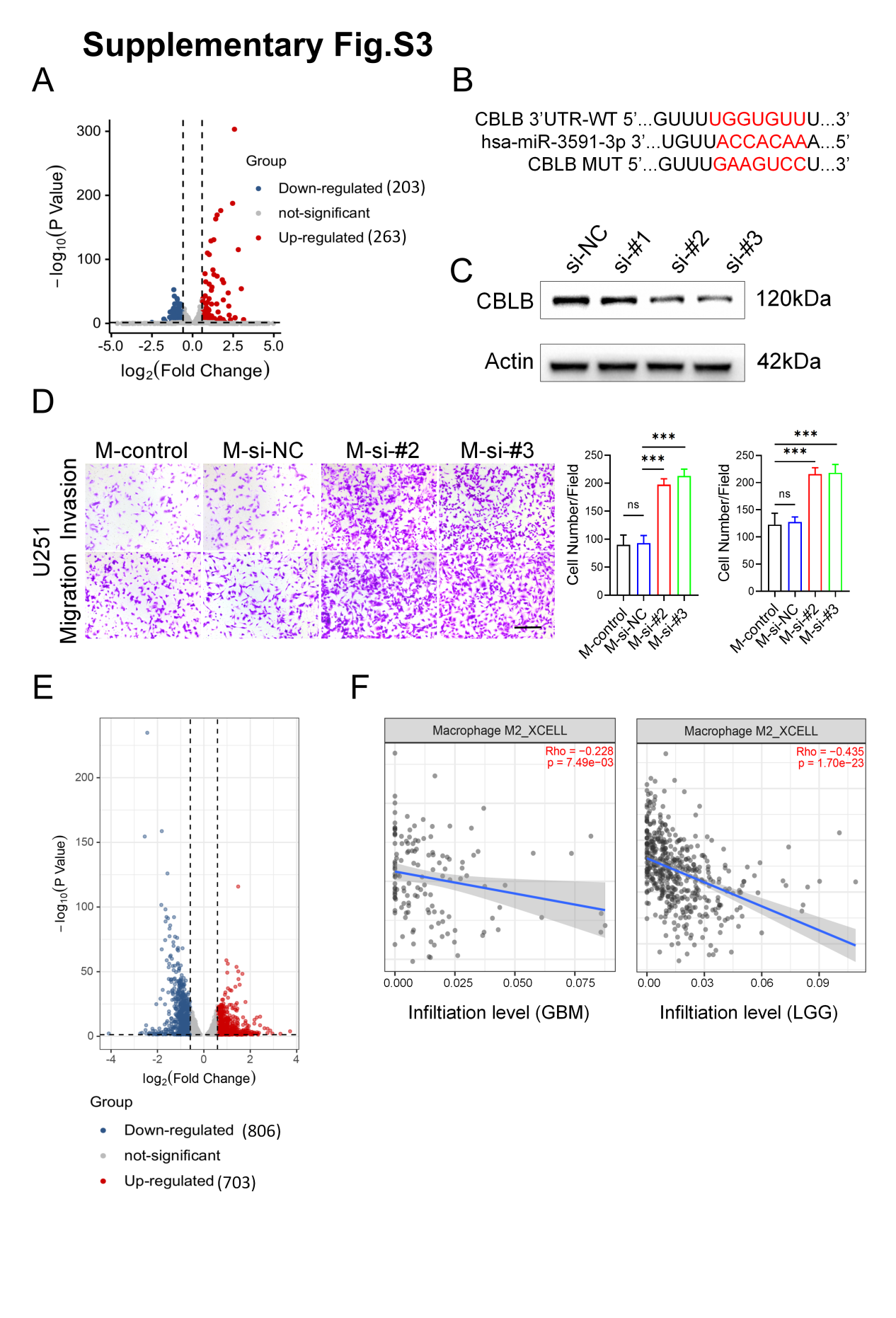
**

**Fig.S3 A** Volcano plot of differentially expressed mRNAs in macrophages treated with miR-3591-3p mimics or miR-NC. **B** Predicted binding sequence of hsa-miR-3591-3p and its binding site in the 3’-UTR of wild-type (WT)/mutant (MUT) CBLB gene. **C** Western blot showing the efficiency of CBLB knockdown in PMA-pretreated THP1 cells. **D** Transwell assay was applied to determine the invasion and migration of U251 cells cocultured with macrophages transfected with si-NC or si-CBLB. Representative images (scale bar, 100 μm) and quantification are shown. **E** Volcano plot of differentially expressed mRNAs in U118MG cells treated with miR-3591-3p mimics or miR-NC. **F** The correlation between the CBLB gene and the infiltration level of M2 macrophages in GBM and LGG was shown in the TIMER (Tumor Immune Estimation Resource) database, respectively. **G** The infiltration levels of immune cell types were quantified in the CBLB-high and CBLB-low groups using Single-Sample Gene Set Enrichment Analysis (ssGSEA). The heatmap shows the enrichment scores of immune cell types between the above two groups. Data are shown as the mean ± SD of three independent experiments. (**p* < 0.05; ***p* < 0.01; ****p* < 0.001).

**
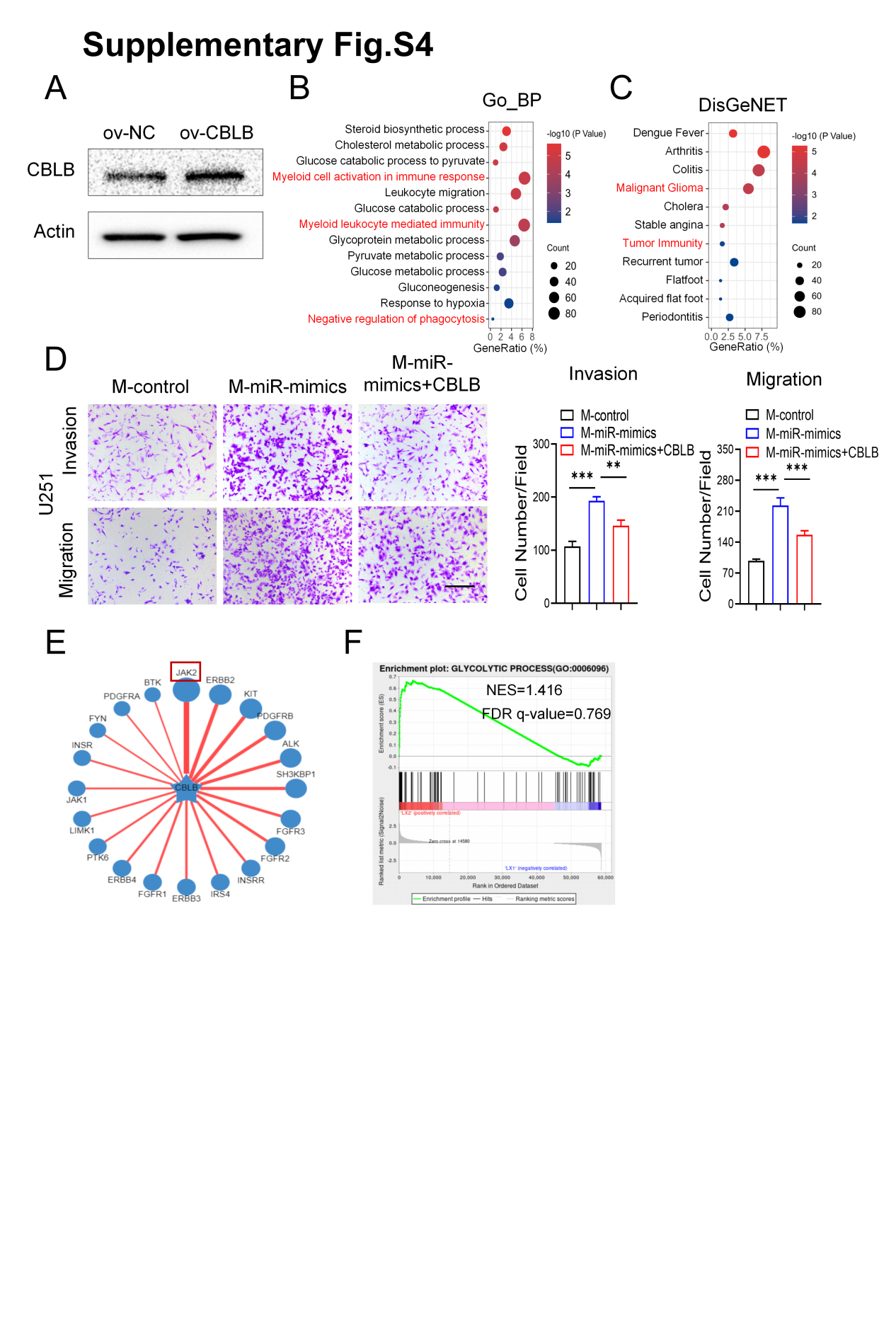
**

**Fig.S4** **A** Overexpression efficiency of CBLB was validated by Western blot. **B** GO (Gene Ontology) enrichment analysis of upregulated mRNA transcripts. **C** The DisGeNet database was used to analyze the association between diseases and upregulated mRNA transcripts. **D** Transwell assay was applied to determine the invasion and migration of U251 cells cocultured with macrophages transfected with miR-3591-3p mimics and CBLB encoding plasmids. Representative images (scale bar, 100 μm) and quantification are shown. **E** UbiBrowser database was used to predict the E3-substrates of CBLB. **F** GO dataset was used for GSEA analysis of mRNA_seq. It showed that differential expression genes in macrophages treated with miR-3591-3p mimics were enriched in the glycolytic process.

**
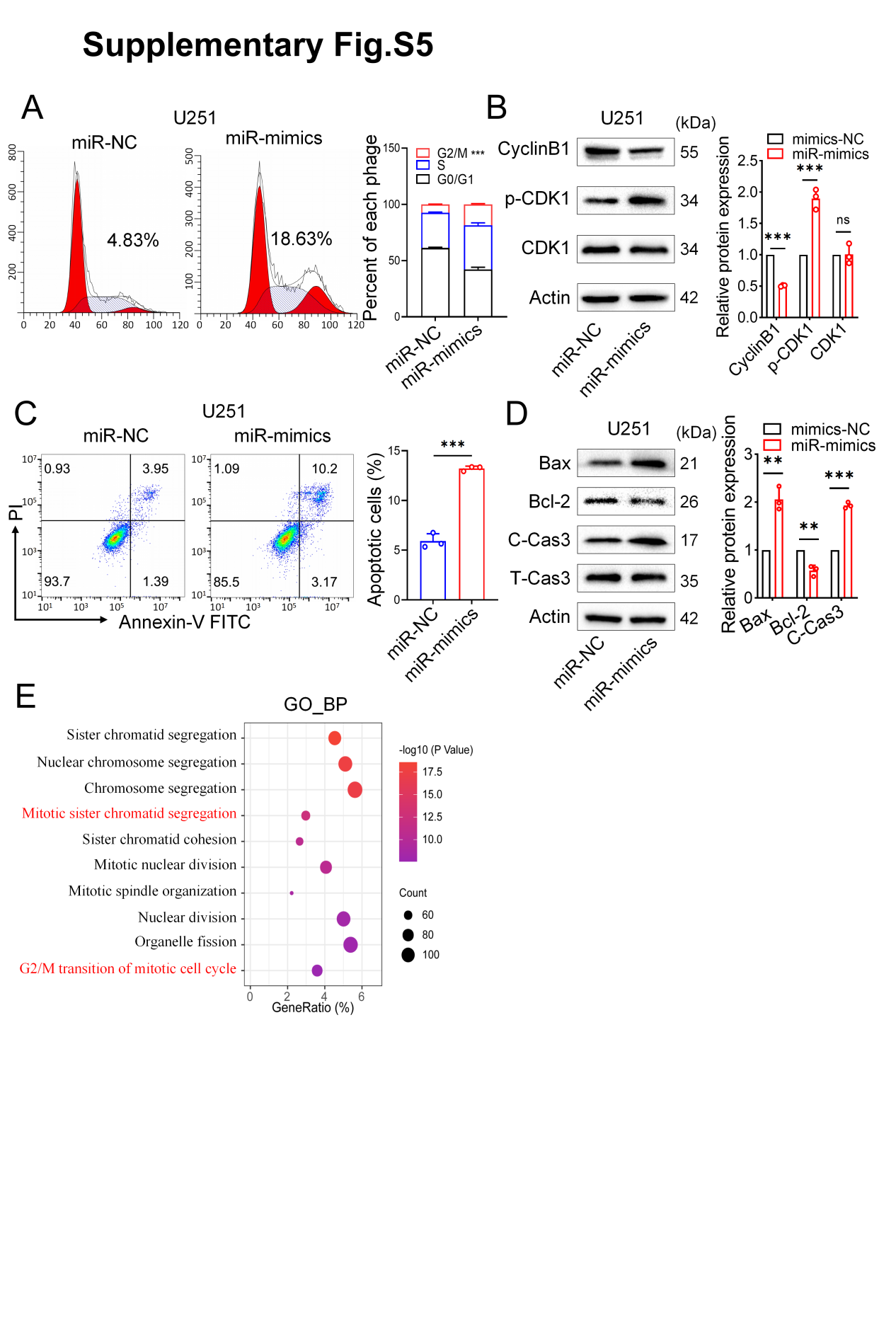
**

**Fig.S5** **Effect of miR-3591-3p on cell cycle and apoptosis in U251 cells.** **A** Flow cytometry analysis of cell cycle transfected with miR-3591-3p mimics or miR-NC in U251 cells. **B** Western blot analysis of G2/M-related proteins in U251 cells treated with miR-3591-3p mimics or miR-NC. **C** Flow cytometry analysis of cell apoptosis transfected with miR-3591-3p mimics or miR-NC in U251 cells. **D** Western blot analysis of apoptosis-associated proteins in U251 cells treated with miR-3591-3p mimics or miR-NC. **E** GO (gene ontology) analysis of down-regulated mRNA based on biological processes in U118MG cells treated with miR-3591-3p mimics. Data are shown as the mean ± SD of three independent experiments. (**p* < 0.05; ***p* < 0.01; ****p* < 0.001).

**
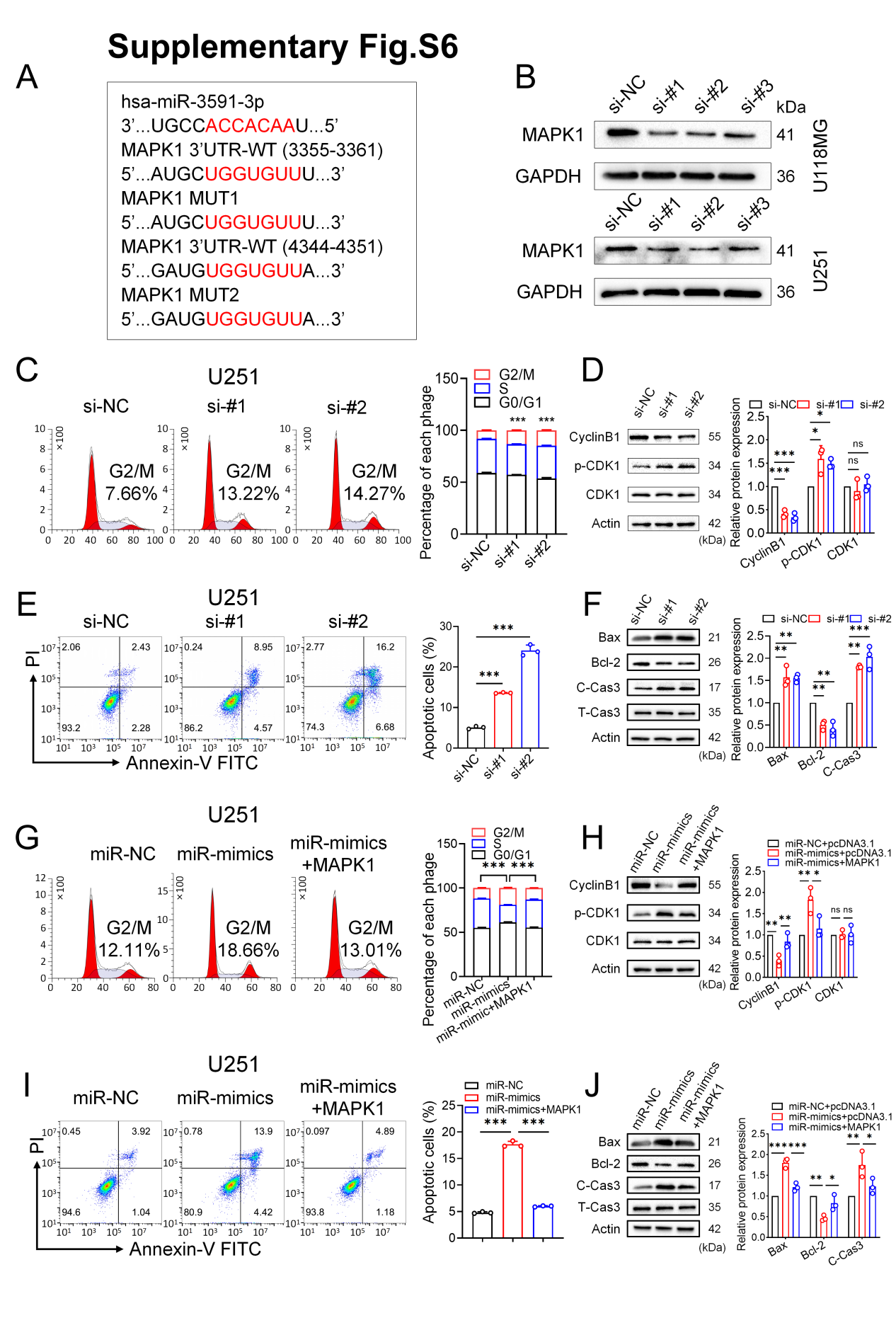
**

**Fig. S6 A** Predicted miR-3591-3p target sequence in the 3′-UTR of MAPK1. The binding site was labeled in red. **B** Efficiency of MAPK1 siRNA knockdown in U118MG and U251 cell lines was evaluated by Western blot analysis. **C** Representative flow cytometry results of the cell cycle phase are shown in U251 cells transfected with si-NC or si-MAPK1. **D** The expression of G2/M-related proteins was detected by Western blot. **E** Representative flow cytometry results of apoptotic cells are shown in U251 cells transfected with si-NC or si-MAPK1. **F** The expression of apoptosis-related proteins was detected by Western blot in groups as indicated. **G** MAPK1 overexpressing plasmids were transfected following miR-3591-3p mimics transfection, and the cell cycle distributions were determined by flow cytometry in U251 cells. **H** The expression of G2/M-related proteins was detected by Western blot in groups as indicated. **I** MAPK1 overexpressing plasmids were transfected following miR-3591-3p mimics transfection, and the apoptotic cells were quantified by flow cytometry in U251 cells. **J** Western blot assessment of the expressions of apoptosis-related proteins in groups as indicated. Data are shown as the mean ± SD of three independent experiments. (**p* < 0.05; ***p* < 0.01; ****p* < 0.001).

**
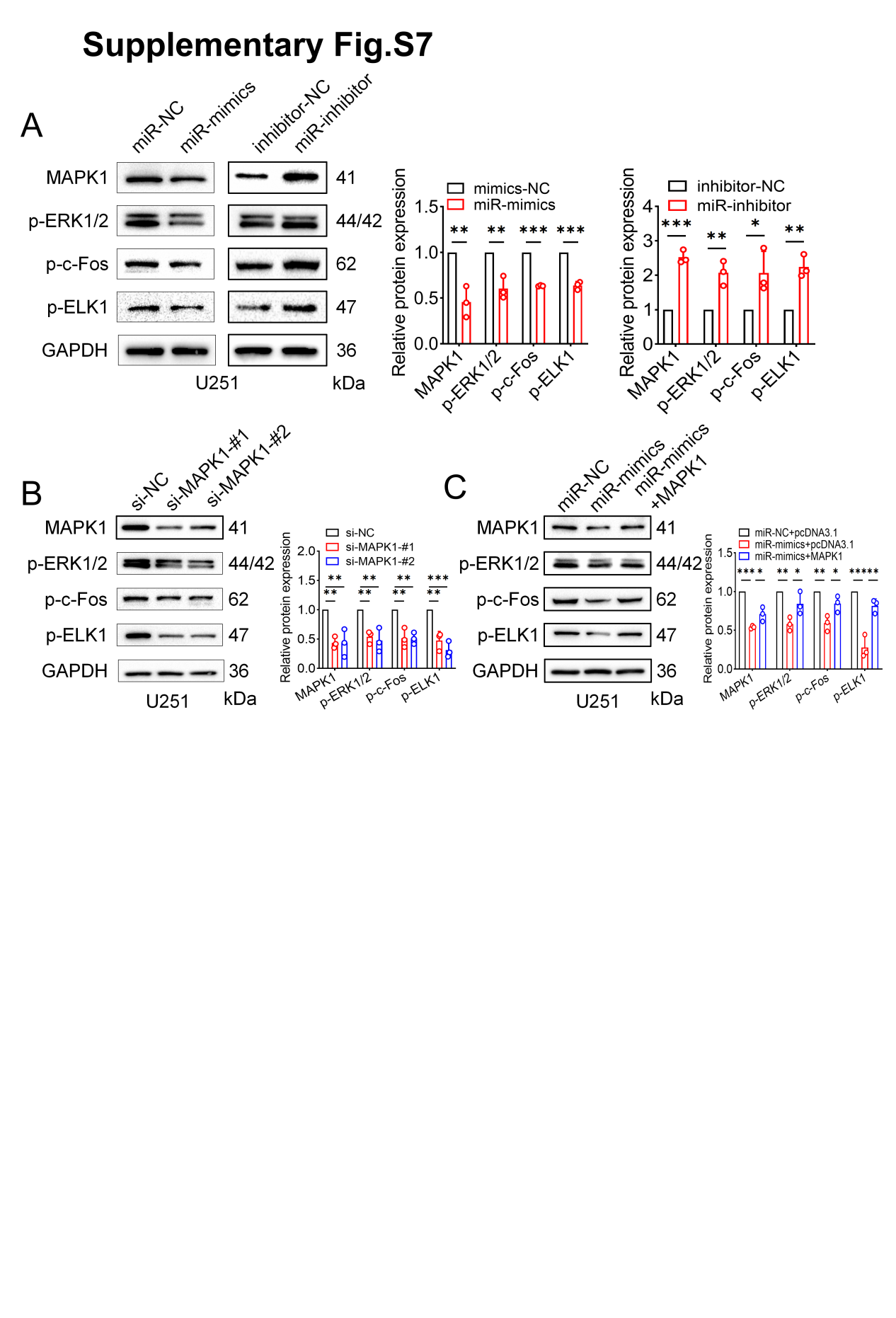
**

**Fig. S7 A** Western blot results for the expression of proteins in the MAPK signaling pathway in U251 cells transfected with miR-NC, miR-3591-3p mimics, inhibitor-NC, and miR-3591-3p inhibitor. **B** Western blot results for the expression of proteins in the MAPK signaling pathway in U251 cells transfected with si-NC or si-MAPK1. **C** MAPK1 overexpressing plasmids were transfected following miR-3591-3p mimics transfection, and the MAPK signaling pathway-related proteins were detected by Western blot in U251 cells. The intensity of protein bands was quantified by densitometry. Data are shown as the mean ± SD of three independent experiments. (**p* < 0.05; ***p* < 0.01; ****p* < 0.001).
