# Supplementary material for: Tumor-derived exosomes deliver the tumor suppressor miR-3591-3p to induce M2 macrophage polarization and promote glioma progression
Source: Oncogene. 2022 Sep 9;41(41):4618–32. doi: 10.1038/s41388-022-02457-w (PMC9546774; doi:10.1038/s41388-022-02457-w)
Supplement: Supplementary file 2 — Table S1 [file 41388_2022_2457_MOESM2_ESM.doc]

| **Table S1 Primers for qRT-PCR** |
| --- |
| Primers for qRT-PCR |
| GAPDH forward: 5′-GCACCGTCAAGGCTGAGAAC-3′ |
| GAPDH reverse: 5′-TGGTGAAGACGCCAGTGGA-3′ |
| U6 forward: 5′-CAGCACATATACTAAAATTGGAACG-3′ |
| U6 reverse: 5′-ACGAATTTGCGTGTCATCC-3′ |
| CBLB forward: 5′-GGGCTCCACTTTTCAGCTCT-3′ |
| CBLB reverse: 5′-GACCATCCGACTCCTGCCAT-3′ |
| DUT forward: 5′-CTGGGTGTTATGGAAGAGTGG-3′ |
| DUT reverse: 5′-GACCATCCGACTCCTGCCAT-3′ |
| CD163 forward: 5′-GGCTTGCAGTTTCCTCAAGA-3′ |
| CD163 reverse: 5′-GACACAGAAATTAGTTCAGCAGCA-3′ |
| IL-10 forward: 5′-GGCACCCAGTCTGAGAACAG-3′ |
| IL-10 reverse: 5′-TGGCAACCCAGGTAACCCTTA-3′ |
| IL-1ra forward: 5′-TGCTACTTTATGGGCAGCAG-3′ |
| IL-1ra reverse: 5′-GGTCGGCAGATCGTCTCTAAA-3′ |
| TGFβ1 forward: 5′-CTGCAAGTGGACATCAACGG-3′ |
| TGFβ1 reverse: 5′-TCCGTGGAGCTGAAGCAATA-3′ |
| Arg-1 forward: 5′-GTGGAAACTTGCATGGACAAC-3′ |
| Arg-1 reverse: 5′-AATCCTGGCACATCGGGAATC-3′ |
| TNFαforward: 5′-CTGCACTTTGGAGTGATCGG-3′ |
| TNFαreverse: 5′-TCAGCTTGAGGGTTTGCTAC-3′ |
| iNOS forward: 5′-CGTGGAGACGGGAAAGAAGT-3′ |
| iNOS reverse: 5′-GACCCCAGGCAAGATTTGGA-3′ |
| miR-3591-3p forward: 5′-GCCGCTTAAACACCATTGTC-3′ |
| miR-3591-3p reverse: 5′-TATGCTTGTTCTCGTCTCTGTGTC-3′ |
| MAPK1 forword:5′-GGCTGTTCCCAAATGCTGAC-3′ |
| MAPK1 reverse:5′-AACTTGAATGGTGCTTCGGC-3′ |
| MAP2K4 forword:5′-ACAGGAGTTCAAAACCCACACA-3′ |
| MAP2K4 reverse:5′-TGCCCACTTGGTTTGTGGAC-3′ |
| MAP2K3 forword:5′-TCAGAGAGGGAGACGTGTGG-3′ |
| MAP2K3 reverse:5′-CAGCACCTTCCGGTCGAACT-3′ |
| FRS2 forword:5′-CATTGGAGGCGAGGGTTTCT-3′ |
| FRS2 reverse:5′-GAACAGGGGTTGGCCCTAAA-3′ |
| DAB2IP forword:5′-TCTCCAACCCCCGAAAACAA-3′ |
| DAB2IP reverse:5′-AAGATCAGGCTCGTTGGCTT-3′ |
| RPS6KA2 forword:5′-TCCCAGTTCACCCAATCGTG-3′ |
| RPS6KA2 reverse:5′-CTTCACGGCATACTCGGTGT-3′ |
| NFATC3 forword:5′-GCCCATTAGGTGGTCCCAAA-3′ |
| NFATC3 reverse:5′-CGGTAGGATGGCTCAAGAGG-3′ |
