# Supplementary material for: Tumor-derived exosomes deliver the tumor suppressor miR-3591-3p to induce M2 macrophage polarization and promote glioma progression
Source: Oncogene. 2022 Sep 9;41(41):4618–32. doi: 10.1038/s41388-022-02457-w (PMC9546774; doi:10.1038/s41388-022-02457-w)
Supplement: Supplementary file 3 — Table S2 [file 41388_2022_2457_MOESM3_ESM.doc]

Table S2. Sequences for siRNAs, microRNA mimics and inhibitors.

| Sequences for siRNA, microRNA mimics and inhibitor |
| --- |
| si-NC (5’ to 3’): sense UUCUCCGAACGUGUCACGUTT  antisense ACGUGACACGUUCGGAGAATT |
| si-CBLB#1:GCCUGAUACAUAUCAGCAUTT  AUGCUGAUAUGUAUCAGGCTT |
| si-CBLB#2:GCGGAAUUGGAAUUUCUUATT  UAAGAAAUUCCAAUUCCGCTT |
| si-CBLB#3:GCGGAAUUGGAAUUUCUUATT  UAAGAAAUUCCAAUUCCGCTT |
| si-MAPK1#1 (5’ to 3’):GCUCCAGAAAUUAUGUUGATT  UCAACAUAAUUUCUGGAGCTT |
| si-MAPK1#2 (5’ to 3’):CCAGGAUACAGAUCUUAAATT  UUUAAGAUCUGUAUCCUGGTT |
| si-MAPK1#3 (5’ to 3’):CAGGGUUCCUGACAGAAUATT  UAUUCUGUCAGGAACCCUGTT |
| miR-3591-3p mimics NC (5’ to 3’):sense UUCUCCGAACGUGUCACGUTTantisense ACGUGACACGUUCGGAGAATT |
| miR-3591-3p mimics (5’ to 3’): AAACACCAUUGUCACACUCCAC  GGAGUGUGACAAUGGUGUUUUU |
| miR-3591-3p inhibitor NC (5’ to 3’): CAGUACUUUUGUGUAGUACAA |
| miR-3591-3p inhibitor (5’ to 3’): GUGGAGUGUGACAAUGGUGUUU |
