# Supplementary material for: Tumor-derived exosomes deliver the tumor suppressor miR-3591-3p to induce M2 macrophage polarization and promote glioma progression
Source: Oncogene. 2022 Sep 9;41(41):4618–32. doi: 10.1038/s41388-022-02457-w (PMC9546774; doi:10.1038/s41388-022-02457-w)
Supplement: Supplementary file 4 — Table S3 [file 41388_2022_2457_MOESM4_ESM.docx]

**Table S3:** Data of antibodies

| **Antibody** | **Catalog number** | **Company** | **MW(kDa)** |
| --- | --- | --- | --- |
| PI3K | #4257 | Cell Signaling Technology | 85 |
| p-PI3K | #4228 | Cell Signaling Technology | 85 |
| AKT | #4691 | Cell Signaling Technology | 60 |
| p-AKT | #4060 | Cell Signaling Technology | 60 |
| mTOR | #2983 | Cell Signaling Technology | 289 |
| p-mTOR | #5536 | Cell Signaling Technology | 289 |
| JAK2 | #3230 | Cell Signaling Technology | 125 |
| p-JAK2 | #3771 | Cell Signaling Technology | 125 |
| STAT3 | #12640 | Cell Signaling Technology | 86 |
| p-STAT3 | #9145 | Cell Signaling Technology | 86 |
| CBLB | Ab32425 | abcam | 120 |
| MAPK1 | 16443-1-AP | proteintech | 41 |
| p-ERK1/2 | #4377 | Cell Signaling Technology | 42,44 |
| c-Fos | #74620 | Cell Signaling Technology | 62 |
| p-c-Fos | #5348 | Cell Signaling Technology | 62 |
| ELK1 | ab32106 | abcam | 47 |
| p-ELK1 | #9181 | Cell Signaling Technology | 47 |
| CyclinB1 | #12231 | Cell Signaling Technology | 55 |
| CDK1 (cdc2) | #28439 | Cell Signaling Technology | 34 |
| p-CDK1 (cdc2) | #4539 | Cell Signaling Technology | 34 |
| Bax | 60267-1-Ig | proteintech | 21 |
| Bcl-2 | #3498 | Cell Signaling Technology | 26 |
| Cleaved Caspase3  (C-Cas3) | 19677-1-AP | proteintech | 17 |
| Total Caspase3  (T-Cas3) | 19677-1-AP | proteintech | 35 |
| CD63 | ab216130 | abcam | 26 |
| TSG101 | ab125011 | abcam | 45 |
| Calnexin | ab133615 | abcam | 90 |
